# Identification of nicotinamide N‐methyltransferase as a promising therapeutic target for sarcopenia

**DOI:** 10.1111/acel.14236

**Published:** 2024-06-05

**Authors:** Rui Liang, Qiao Xiang, Miao Dai, Taiping Lin, Dongmei Xie, Quhong Song, Yu Liu, Jirong Yue

**Affiliations:** ^1^ Department of Geriatrics and National Clinical Research Center for Geriatrics, West China Hospital Sichuan University Chengdu China; ^2^ Department of Geriatrics Jiujiang No 1 People's Hospital Jiujiang Jiangxi China; ^3^ National Clinical Research Center for Geriatrics, General Practice Ward/International Medical Center Ward, General Practice Medical Center, State Key Laboratory of Biotherapy, West China Hospital Sichuan University Chengdu China

**Keywords:** sarcopenia, NNMT, NAD^+^, metabolic dysregulation, diagnostic biomarker

## Abstract

Sarcopenia is a significant geriatric syndrome that involves the loss of skeletal muscle mass and strength. Due to its substantial endocrine role, the metabolic microenvironment of skeletal muscle undergoes changes with age. Examining the pathogenesis of sarcopenia through focusing on metabolic dysregulation could offer insights for developing more effective intervention strategies. In this study, we analyzed the transcriptomics data to identify specific genes involved in the regulation of metabolism in skeletal muscle during the development of sarcopenia. Three machine learning algorithms were employed to screen key target genes exhibiting strong correlations with metabolism, which were further validated using RNA‐sequencing data and publicly accessible datasets. Among them, the metabolic enzyme nicotinamide N‐methyltransferase (NNMT) was elevated in sarcopenia, and predicted sarcopenia with an area under the curve exceeding 0.7, suggesting it as a potential therapeutic target for sarcopenia. As expected, inhibition of NNMT improved the grip strength in aging mice and alleviated age‐related decline in the mass index of the quadriceps femoris muscles and whole‐body lean mass index. Additionally, the NNMTi treatment increased the levels of nicotinamide adenine dinucleotide (NAD^+^) content, as well as PGC1α and p‐AMPK expression in the muscles of both the D‐galactose‐treated mouse model and naturally aging mouse model. Overall, this work demonstrates NNMT as a promising target for preventing age‐related decline in muscle mass and strength.

## Graphical abstract

(a) The etiology of sarcopenia encompasses various factors that synergistically contribute to age‐related damages, leading to metabolic dysregulation and subsequent reductions in muscle strength and mass. (b) We integrated transcriptome data from three cohorts of sarcopenia individuals, applying machine learning algorithms to identify metabolic target genes. Subsequent validation in additional muscle datasets confirmed NNMT as a noteworthy metabolic target of sarcopenia with diagnostic potential. Targeted intervention in the NNMT gene may modulate NAD^+^‐related pathways to improve metabolic damage of muscle tissues.

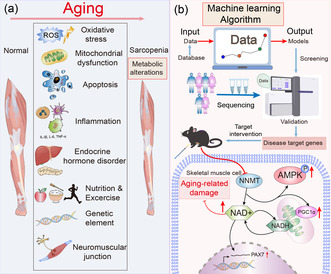

**Abbreviations**
ADH1B: Alcohol dehydrogenase 1B
AMPK: Adenosine monophosphate‐activated protein kinase
AUC: Area under the curve
CERS3: Ceramide synthase 3
CYP26B1: Cytochrome P450 family 26 subfamily B member 1
DEG: Differential expressed gene
D‐gal: D‐galactose
DO: Disease Ontology
DXA: Dual X‐ray absorptiometry
FOXO1: Forkhead box O1
Foxo3: Forkhead box O3
GA: Gastrocnemius
GEO: Gene Expression Omnibus
GO: Gene Ontology
GSEA: Gene set enrichment analysis
GTEx: Genotype‐Tissue Expression
Ho‐1: Heme oxygenase 1
KEGG: Kyoto Encyclopedia of Genes and Genomes
LASSO: Least absolute shrinkage and selection operator
LMI: Lean mass index
MHC: Major histocompatibility complex
MNAM: N1‐methylnicotinamide
MS: Metabolic syndrome
MurF1: Muscle ring finger protein‐1
MYH1: Myosin heavy chain 1
Myh2: Myosin heavy chain 2
Myh7: Myosin heavy chain 7
MYOD1: Myogenic differentiation 1
NAD+: Nicotinamide adenine dinucleotide
NAM: Nicotinamide
Nampt: Nicotinamide phosphoribosyltransferase
NMN: Nicotinamide mononucleotide
Nmnat1: Nicotinamide nucleotide adenylyltransferase 1
NNMT: Nicotinamide N‐methyltransferase
NNMTi: NNMT inhibitor
Nrf2: Nuclear respiratory factor
p‐AMPK: Phospho‐adenosine monophosphate‐activated protein kinase
Pax7: Paired box 7
p‐FOXO1: Phospho‐forkhead box O1
PGC1α: Peroxisome proliferator‐activated receptor‐gamma coactivator‐1 alpha
PNPLA3: Patatin like phospholipase domain containing 3
PPARγ: Peroxisome proliferator activated receptor γ
PRKAR2B: Protein kinase cAMP‐dependent type II regulatory subunit beta
PX: ProteomeXchange
QF: Quadriceps femoris
qPCR: Quantitative real‐time polymerase chain reaction
RNA‐seq: RNA sequencing
ROC: Receiver operating characteristic
ROS: Reactive oxygen species
SAH: S‐adenosyl homocysteine
SAM: S‐adenosylmethionine
Sirt1: Sirtuin 1
Sirt3: Sirtuin 3
SMI: Skeletal muscle mass index
SVM‐RFE: Support vector machine recursive feature elimination
TA: Tibialis anterior
TGFβ: Transforming growth factor beta 1

## 1 INTRODUCTION

Over the past few decades, advancements in healthcare technology and continuous socioeconomic development have led to a rapid increase in life expectancy and the proportion of the older population. By 2050, the number of adults aged 65 years and older worldwide will reach 1.6 billion (Nations, 2022). Sarcopenia, a significant geriatric syndrome that affects muscle mass and/or strength, leads to somatic dysfunction (Cruz‐Jentoft et al., 2010). Its prevalence ranges from 10% to 27% in individuals over 60 years of age and can exceed 50% in some care institutions of older adults (Papadopoulou et al., 2020; Petermann‐Rocha et al., 2022). Sarcopenia poses a serious threat to the physical and mental health of older adults, increasing the risk of falls, fractures, disability, and even death (Cruz‐Jentoft et al., 2010). Additionally, it serves as a risk factor for adverse outcomes of multiple chronic diseases, including diabetes and cardiovascular disease (Liccini & Malmstrom, 2016; Wathanavasin et al., 2022), imposing heavy economic and caregiving burdens on patients' families and society. Despite receiving significant attention, the exact pathogenesis of sarcopenia remains unclear.

Skeletal muscle accounts for approximately 40% of the human body mass, serving as a producer of multiple hormones and playing a crucial role in glycolipid metabolism and hormone function (Yang & Chan, 2022). Muscle aging is commonly associated with alterations in the metabolic microenvironment, such as changes in energy and cell homeostasis pathways and shifts in the levels of certain transcription factors, even during the pre‐sarcopenia stage (Yang & Chan, 2022). Dysregulated metabolism is a critical pathological feature in the skeletal muscles of patients with sarcopenia, directly affecting the synthesis and breakdown of proteins, glycogen, and fats in muscle tissue (Daily & Park, 2022; Shoemaker et al., 2022). Consequently, this impairs energy metabolism, muscle strength, and the regenerative capacity of the muscles.

Nicotinamide N‐methyltransferase (NNMT), an important metabolic enzyme responsible for catalyzing the transfer of a methyl group from the donor S‐adenosyl methionine (SAM) to nicotinamide (NAM), plays a key role in regulating nicotinamide adenine dinucleotide (NAD^+^) metabolism and DNA methylation status (Roberti et al., 2021). It is notably abundant in various tissues, including the liver, fat, and skeletal muscle. The dysregulation of NNMT expression is closely associated with the risk of metabolic diseases such as obesity and diabetes (Liu et al., 2021). Additionally, NNMT functions as a critical regulator in certain tumors, with implications for tumor malignancy and unfavorable prognosis (Wang et al., 2022). Therefore, NNMT has been demonstrated as a promising target for the treatment of some metabolic diseases and tumors (Liu et al., 2021; Wang et al., 2022). However, limited research has explored the role of NNMT in sarcopenia, particularly its involvement in the metabolic dysregulation of aging skeletal muscles.

Current interventions for sarcopenia primarily involve rehabilitation and nutritional supplementation, as there are no approved medications with a consensus (Liu & Yue, 2022; Mellen et al., 2023). Metabolic dysregulation plays a significant role in the pathogenesis and progression of sarcopenia. This study aims to identify the potential metabolism‐related genes involved in the regulation of sarcopenia and muscle aging by widely analyzing the transcriptomic data from both publicly available datasets and our own. Notably, NNMT plays a critical role in the metabolic dysregulation of aging skeletal muscles and may serve as a potential biomarker for sarcopenia diagnosis. Targeted inhibition of NNMT improves the energy metabolism of skeletal muscles and alleviates muscle damage caused by metabolic disorders through regulating the NAD^+^/AMPK axis in both the D‐galactose‐treated mouse model and the naturally aging mouse model. This study sheds light on the diagnostic and therapeutic values of NNMT in sarcopenia, opening new avenues for exploring pharmacological treatment strategies for sarcopenia.

## 2 MATERIALS AND METHODS

### 2.1 Data acquisition and preprocessing

To increase the sample size and enhance the analysis robustness, we integrated multiple datasets from the Gene Expression Omnibus (GEO) database (https://www.ncbi.nlm.nih.gov/geo/), including three RNA‐sequencing datasets (GSE111006, GSE111010, and GSE111016) and two microarray datasets (GSE38718 and GSE8479). To address any potential batch effects, we used the comBat_seq or comBat function, available in the R package “sva” (Leek et al., 2012; 2014), to perform batch effect correction. As a result, Dataset_1 (RNA‐sequencing data) and Dataset_2 (microarray data) were obtained. Detailed information is recorded in Table S1.

### 2.2 Analysis of differential expressed genes (DEGs)

The analysis of DEGs for Dataset_1 and Dataset_2 was conducted separately using the “limma” package (Ritchie et al., 2015) with appropriate filtering criteria (absolute log_2_ [Fold change] >0.585 and *p* < 0.05). To gain insights into the functional implications of the DEGs from Dataset_1, Kyoto encyclopedia of genes and genomes (KEGG) pathways, Gene Ontology (GO) enrichment, and Disease Ontology (DO) analysis were performed using R package “ggplot2,” “enrichplot,” “DOSE,” and “clusterProfiler.” Additionally, Gene set enrichment analysis (GSEA) was conducted using the “GSEABase” package based on “c2.cp.kegg.v7.5.1.symbols.gmt” dataset in Dataset_1. The enrichment terms of the DEGs were also explored using Metascape analysis (https://metascape.org/).

### 2.3 Screening metabolic DEGs

The metabolic gene dataset was acquired from the Pathway Unification Database (https://pathcards.genecards.org/), and metabolic DEGs were obtained by performing overlapping analysis. The R package “glmnet,” “randomForest,” “e1071,” and “caret” were used for least absolute shrinkage and selection operator (LASSO) regression, Random forest, and support vector machine recursive feature elimination (SVM‐RFE) analysis of the metabolic DEGs. The selected genes were then subjected to receiver operating characteristic (ROC) analysis. To further refine the selection, we screened for genes with the area under the curve (AUC) values >0.7 using Dataset_1. The expression levels of the selected genes, along with their respective AUC values, were subsequently validated in Dataset_2.

### 2.4 Muscle tissue collection and transcriptome sequencing

Patients with sarcopenia were identified according to the Asian Working Group for Sarcopenia 2019 criteria to simultaneously satisfy the diagnostic standards of grip strength (Jamar hand‐held dynamometer, Sammons Preston, USA) and skeletal muscle mass index (bioelectrical impedance analyzer, InBody S10, Korea). Non‐sarcopenia was defined as not meeting either of these criteria. Ten older adults participated in the study, with five diagnosed with sarcopenia and five without sarcopenia. All participants were informed about the study and provided signed informed consent forms. Approximately 2 cm^3^ of muscles of the lower limb were taken from each person under general anesthesia (further details are presented in Data S1—2.1). Ethical approval for the study was obtained from the Ethics Committee on Biomedical Research of West China Hospital of Sichuan University (approval No: 2020‐539).

Total RNA was extracted from muscle tissues using the TRIzol method. RNA purity and integrity were checked using a NanoPhotometer (Implen) and an Agilent 2100 bioanalyzer (Agilent Technologies), respectively. Sequencing was conducted on the Illumina NovaSeq 6000 platform with paired‐end 150‐bp reads and the raw counts were transformed into TPM values (Dataset_3) using R software. DEGs, KEGG, and GO analyses were conducted using the methods described in 2.2.

### 2.5 Relationship analysis between NNMT and sarcopenia

The expression levels of NNMT in different human tissues and their changes with age were analyzed using the Genotype‐Tissue Expression (GTEx) database. Differences in expression levels of NNMT between young and aged muscles were explored using the data from GEO database (GSE164471, GSE175495, and GSE136344) and the ProteomeXchange (PX) Consortium (PXD011967). Besides, GSE126101 and GSE85718 were downloaded and used to investigate the impacts of TNF‐α, palmitate, or nicotinamide mononucleotide (NMN) treatment on the expression level of NNMT (Details of the data sets are presented in Data S1; Table S1).

### 2.6 Animal experiments

Eight‐week‐old male C57BL6 mice were used to construct a D‐galactose (D‐gal; Solaibao, China)‐induced aging mouse model. Briefly, mice were subcutaneously injected with D‐gal (150 mg/kg, bid) or saline for 5 weeks, and intraperitoneally injected with 5 or 10 mg/kg/d NNMT inhibitor (NNMTi, HY‐131042, MCE MedchemExpress, USA) or an equal volume of blank solvent prepared according to the manufacturer's instructions. Nineteen‐month‐old female C57BL6 mice (*n* = 10) were randomly assigned to two groups. Following an adaptive culture period, the mice were intraperitoneally administered either 20 mg/kg/d NNMTi or an equivalent volume of saline for 5 weeks. According to the manufacturer's instructions, whole‐body lean mass and fat mass were assessed using dual x‐ray absorptiometry (iNSiGHT VET DXA, OsteoSys, Korea) on the first and last day of drug administration. All mice were obtained from Beijing Vital River Laboratory Animal Technology Co., Ltd.

The grip force of mice was regularly measured using a Grip strength meter (SA417, Jiangsu Science Biological Technology Co.Ltd), and the changes in the grip index (grip force/weight) were calculated from the baseline. The quadriceps femoris (QF), gastrocnemius (GA), and tibialis anterior (TA) muscles were detached and weighed. Animal ethics approval was acquired from the Animal Ethics Committee of West China Hospital of Sichuan University (No. 20220929001).

### 2.7 Muscle contractility measurement

Muscle contraction forces were measured in old mice using a biological function experimental system (BL‐420N, Tai Meng Technology Co., Ltd., Chengdu, China). The whole procedure was performed according to the manufacturer's instructions and the established protocols reported (Lafoux et al., 2020). In short, the intact GA muscle was dissected and attached to a force transducer. Stimulation electrodes were placed at the muscle's midpoint and connected to a pulse generator. The muscle was stretched and the stimulation voltage (0.5–4 V stimulation amplitude, increments of 0.05 V) was adjusted to achieve maximum contraction force. The same procedure was repeated on the QF muscle with the mouse repositioned supine. Force‐voltage relationships for both muscles were then analyzed to evaluate their contraction response.

### 2.8 Measurement of NAD ^+^ content

The NAD^+^ content and NAD^+^/NADH ratio in the QF muscle were measured using an NAD^+^/NADH assay kit with WST‐8 (Beyotime Biotechnology, China). According to the manufacturer's instructions, approximately 20 mg of muscle tissue from each mouse was weighed, minced, and lysed with 400 μL of lysis buffer. To measure the total NAD^+^/NADH content, 20 μL of supernatant and 90 μL of alcohol dehydrogenase were incubated at 37°C for 10 min, followed by the addition of chromogenic solution and a further incubation for 30 min. Meanwhile, another 20 μL of supernatant was incubated at 60°C for 30 min to decompose NAD^+^, and then chromogenic solution was added to measure the NADH content. The absorbance of the reaction solution was measured at 450 nm. A standard curve was generated to calculate the NADH content in the samples. The NAD^+^ content was obtained by subtracting the NADH content from the total NAD^+^/NADH content, and the amount was normalized to the tissue weight.

### 2.9 Molecular docking

The 3D structure of NNMTi was drawn using Chem3D software (Ultra 14.0, CambridgeSoft, USA). The protein structure of NNMT (PDB ID: 6PVS) was obtained from the Protein Data Bank (https://www.rcsb.org/). The virtual docking between NNMTi and 6PVS was performed using the AutoDock Vina method, with the docking center at 1.964 Å × −0.178 Å × −7.627 Å (Trott & Olson, 2010). The best docking image was drawn using PyMOL (version 1.7.2.1).

### 2.10 Statistical analysis

Statistical analyses were performed using R software (version 4.0.2) and GraphPad Prism 8 (version 8.4.0). RandomForest and SVM‐RFE algorithms were calculated using R 4.2.1. Numerical data are presented as the mean and standard deviation (SD) or standard error of the mean (SEM). Categorical data are expressed as frequencies and percentages. A *t*‐test or analysis of variance (ANOVA) was conducted for parametric values. Wilcoxon‐ or Mann–Whitney test was used to analyze nonparametric values. A *p* value <0.05 was considered statistically significant.

### 2.11 Additional methods

Additional details of Materials and methods are described in Data S1.

## 3 RESULTS

### 3.1 Sarcopenia is closely related to metabolic dysregulation

**FIGURE 1:**
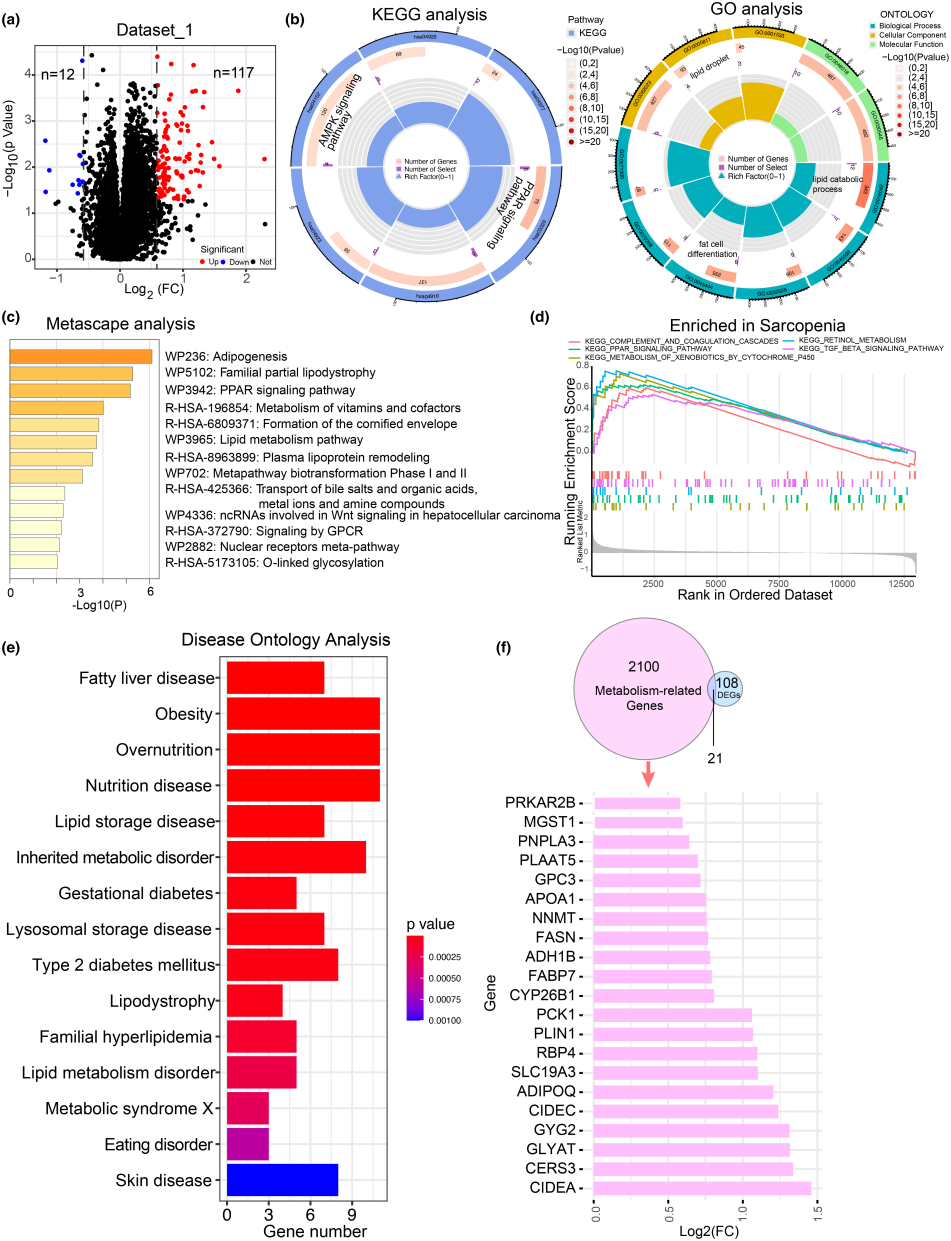
Dysregulated metabolism in sarcopenia. (a) Volcano plots of DEGs absolute log_2_ (ratio [sarcopenia/normal]) >0.585 and *p* < 0.05, based on “limma” R package) in Dataset_1 which was obtained through merging three GEO datasets. Red indicates genes upregulated in sarcopenia, and blue represents downregulated genes. (b) GO and KEGG enrichment analysis of DEGs showing in (a). (c–e) Enrichment analysis of DEGs by Metascape (c), GSEA (d) and Disease ontology (e). (f) Venn diagram presented the intersection of metabolism‐related gene set with DEGs. Bar chart showed that the relative expression levels of 21 metabolic genes were upregulated in sarcopenia.

A total of 129 DEGs were identified by analyzing the transcriptome data obtained from the GEO database (Dataset_1, Normal: *n* = 62, Sarcopenia: *n* = 33, Figure S1). KEGG and GO analyses showed that DEGs were mainly enriched in PPAR and AMPK signaling pathways and associated with lipid catabolic process and lipid droplet terms (Figure 1a,b). Further analysis using Metascape indicated that the DEGs were related to adipogenesis, familial partial lipodystrophy, lipid metabolism pathways, and the metabolism of vitamins and cofactors. Besides, GESA revealed that sarcopenia‐associated pathways also included retinol metabolism, metabolism of xenobiotics by cytochrome P450 and TGFβ signaling pathways. Moreover, disease ontology analysis revealed that the sarcopenia‐associated DEGs had potential roles in fatty liver disease, obesity, metabolic disorders, and diabetes (Figure 1e). These results collectively suggested the association between metabolic regulation and sarcopenia. Therefore, we performed an intersection analysis between the DEGs and the metabolic geneset obtained from the Pathway Unification Database, resulting in the identification of 21 metabolic genes. Notably, all of these genes were found to be upregulated in sarcopenia, and many of them were associated with energy or lipid metabolism, like protein kinase cAMP‐dependent type II regulatory subunit beta (PRKAR2B), patatin like phospholipase domain containing 3 (PNPLA3), NNMT and alcohol dehydrogenase 1B (ADH1B) (Figure 1f).

### 3.2 NNMT is a crucial metabolic gene involved in sarcopenia

**FIGURE 2:**
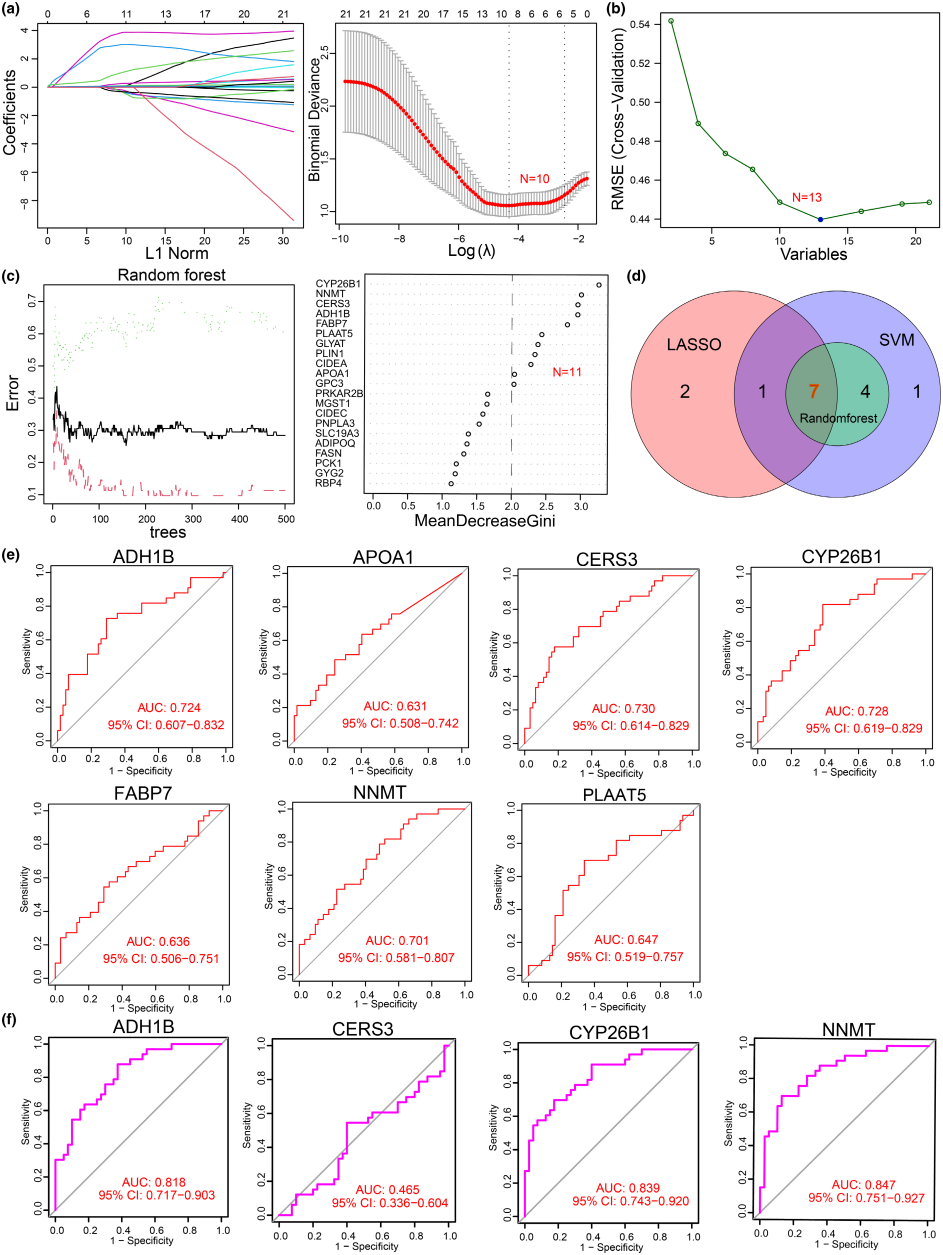
Screening of the metabolism‐related genes in sarcopenia. (a) Ten target genes were identified out of the 21 metabolic genes using least absolute shrinkage and selection operator (LASSO) regression with tenfold cross‐validation. (b) Target gene selection via support vector machine recursive feature elimination (SVM‐RFE) algorithm. (c) Eleven target genes were obtained by Random Forest algorithm with importance score greater than 2. (d) Venn diagram shows the overlapping of genes screened by the three algorithms. (e, f) Receiver operating characteristic (ROC) analysis for target genes in Dataset_1 (e) and Dataset_2 (f).

To further reveal the most critical metabolic genes associated with sarcopenia, the above 21 metabolic genes were used for LASSO regression, Random Forest, and SVM‐RFE analyses, and 10, 11, and 13 genes were considered as feature selections, respectively (Figure 2a–c; Figures S2–S4). From the intersection of these three screening approaches, a set of seven metabolic genes was identified. Then, ROC analysis was conducted to assess the diagnostic value of the target genes in sarcopenia, and the AUC values of CERS3, ADH1B, CYP26B1, and NNMT were greater than 0.7 (Figure 2e). Further analysis using Dataset_2 showed that the AUC values of the ADH1B, CYP26B1, and NNMT were above 0.7, specifically, 0.818 (95% CI, 0.717–0.903) for ADH1B, 0.839 (95% CI, 0.743–0.920) for CYP26B1, and 0.847 (95% CI, 0.717–0.903) for NNMT. However, only the expression level of NNMT was significantly upregulated in the old muscle group compared to the young muscle group, indicating its potential role in the regulation of the age‐related sarcopenia (Figure 2f; Figure S5a).

**FIGURE 3:**
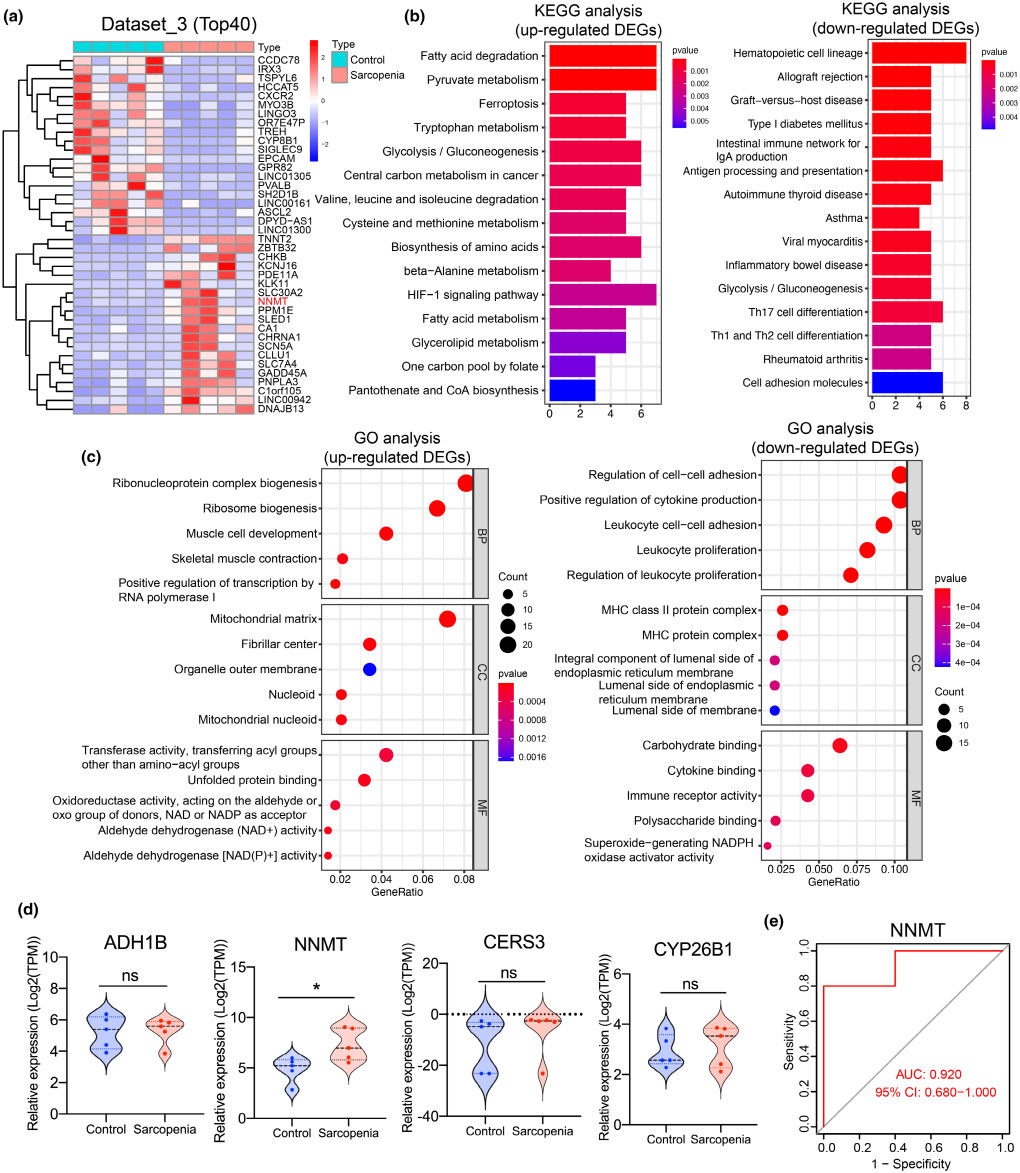
RNA sequencing (RNA‐seq) analysis and the verification of the target genes (Dataset_3). (a) Heat map presenting the top 40 differentially expressed genes (DEGs, absolute log_2_ ratio [sarcopenia/normal]) >0.585 and *p* < 0.05) of RNA‐seq analysis of muscle tissues from patients with or without sarcopenia (Dataset_3). (b, c) KEGG pathways (b) and GO enrichment analysis (c) for up and downregulated DEGs. (d) Relative expression levels of target genes (Dataset_3). *p < 0.05, tested by “limma” R package. (e) ROC analysis for NNMT.

To further validate the above findings, 10 muscle samples (5 normal and 5 sarcopenia) were collected and subjected to RNA‐sequencing (Dataset_3; Table S1), and 545 DEGs were identified (Figure 3a). Upon conducting KEGG and GO analyses, we observed that the 325 upregulated genes were mainly enriched in multiple metabolism‐related pathways including fatty acid degradation, pyruvate metabolism, and glycolysis/gluconeogenesis. Additionally, GO terms were related to energy metabolism, such as mitochondrial matrix and aldehyde dehydrogenase (NAD^+^) activity. On the other hand, the main enriched pathways of the 220 downregulated genes were also related to metabolisms, such as type I diabetes mellitus and glycolysis/gluconeogenesis pathways, and GO terms consisted of the MHC protein complex and carbohydrate binding (Figure 3b,c). Notably, NNMT expression was also significantly upregulated in the sarcopenia group in Dataset_3, with an AUC value of 0.92 (Figure 3d,e). Overall, these results collectively indicate that dysregulation of NNMT may be closely associated with sarcopenia.

### 3.3 NNMT is involved in the aging process and metabolic dysregulation in skeletal muscle

**FIGURE 4:**
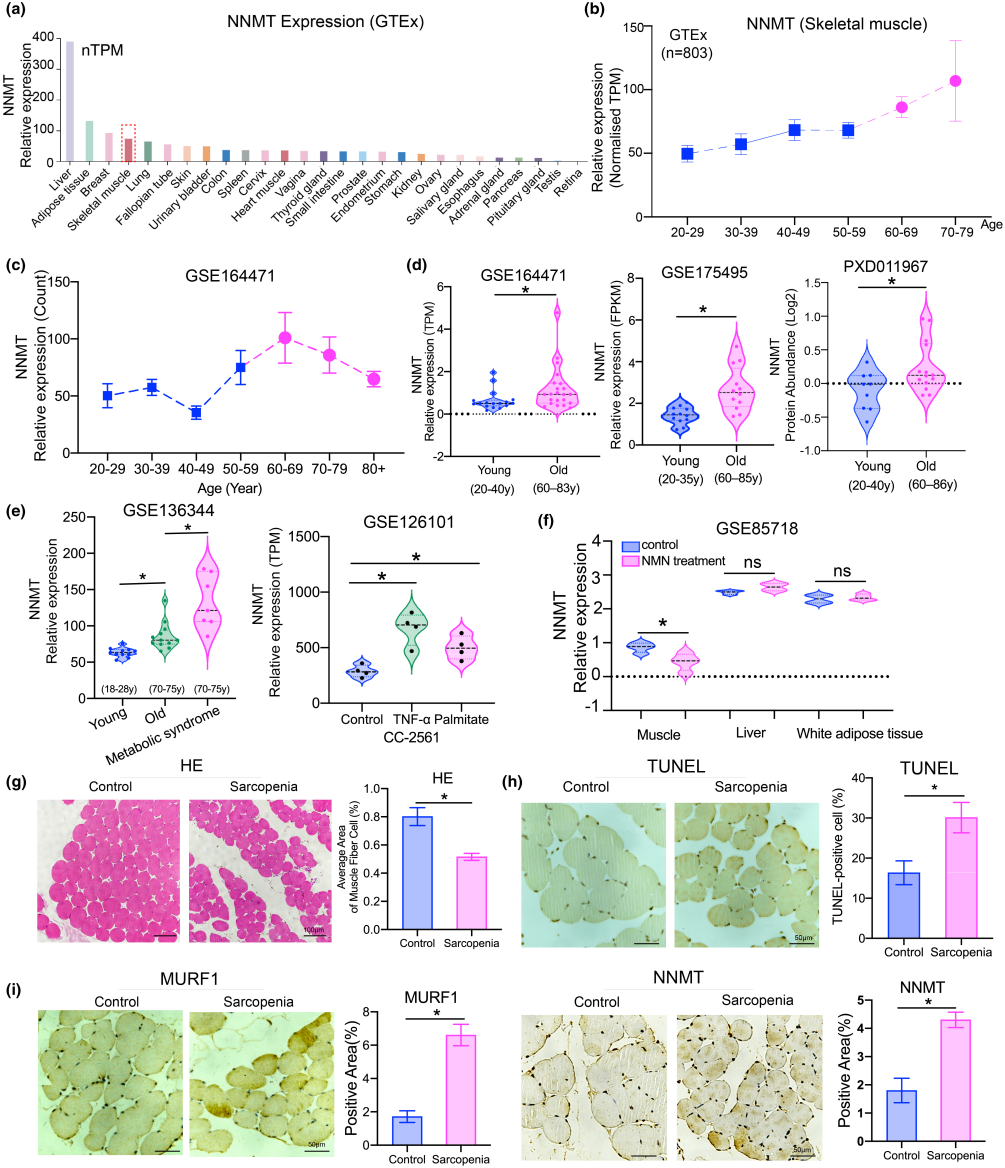
Expression level of NNMT changes with aging and metabolic dysregulation in muscles. (a) Expression levels of NNMT in different human tissues from GTEx database. (b, c) NNMT mRNA expression levels in skeletal muscles change with age based on analyzing GTEx data (b) and GSE164471 dataset (c). (d) Analyses of GEO database (GSE164471, GSE175495 and GSE136344 datasets) and ProteomeXchange (PX) Consortium (PXD011967) presented the different mRNA and protein expression levels of NNMT in young and old skeletal muscle. (e) Analysis of GSE136344 data suggested that the expression levels of NNMT in skeletal muscles of the young people and older adults with or without metabolic dysregulation. GSE126101 data supported that NNMT expression in skeletal cell (CC‐2561) was upregulated by TNF‐α or palmitate treatment. (f) Effects of NMN treatment on NNMT expression level in muscle, liver, and white adipose tissues. (g) Typical images presenting HE staining for myofiber cross‐section of skeletal muscle. (h) Analysis of apoptosis in skeletal muscle using TUNEL staining. The proportion of TUNEL‐positive cells were measured using ImageJ. (i) Representative images on IHC staining for expression levels of MURF1 and NNMT in skeletal muscles. Student *t*‐test or analysis of variance (ANOVA) was conducted for parametric values. Mann–Whitney test was used to analyze nonparametric values. *p < 0.05, ns, not significant.

According to data from the GTEx database, NNMT showed relatively high expression levels in the human liver, fat, and skeletal muscles (Figure 4a; Figure S5b), and its expression levels increased with age in skeletal muscles (Figure 4b–d). NNMT is intricately linked to both skeletal muscle aging and metabolic dysregulation. Analysis of the GSE136344 dataset revealed that muscles from healthy old people had higher NNMT expression levels than those of young people, while exhibiting lower NNMT expression levels than those of old patients with metabolic syndrome. Additionally, by analyzing the GSE126101 dataset, it revealed that the stimulation with TNF‐α or palmitate significantly affected gene expressions associated with skeletal muscle dysfunction and metabolic dysregulation, upregulating the expression of numerous metabolic genes in skeletal muscle cells (CC‐2561), including NNMT (Figure 4e). NMN serves as an effective NAD^+^ supplement, which is crucial for metabolic functions such as maintaining glucose and insulin homeostasis and improving mitochondrial respiratory capacity in aging muscles (Mills et al., 2016). Analysis of the GSE85718 dataset suggested that NMN treatment for 12 months upregulated the NAD^+^ content in tissues, while reducing the expression levels of NNMT in the skeletal muscles of C57BL/6N mice. There was no significant impact on NNMT expression in the liver or white fat (Figure 4f). In patients with sarcopenia, there was a slight reduction in the mean myofiber cross‐sectional area and a significant increase in the proportions of apoptotic cells. Immunohistochemical (IHC) staining revealed that the expression levels of muscle ring finger protein‐1(MurF1) and NNMT were higher in the sarcopenia group than in the non‐sarcopenic group (Figure 4g–i).

### 3.4 Targeted intervention of NNMT ameliorates aging‐associated damage of skeletal muscle

**FIGURE 5:**
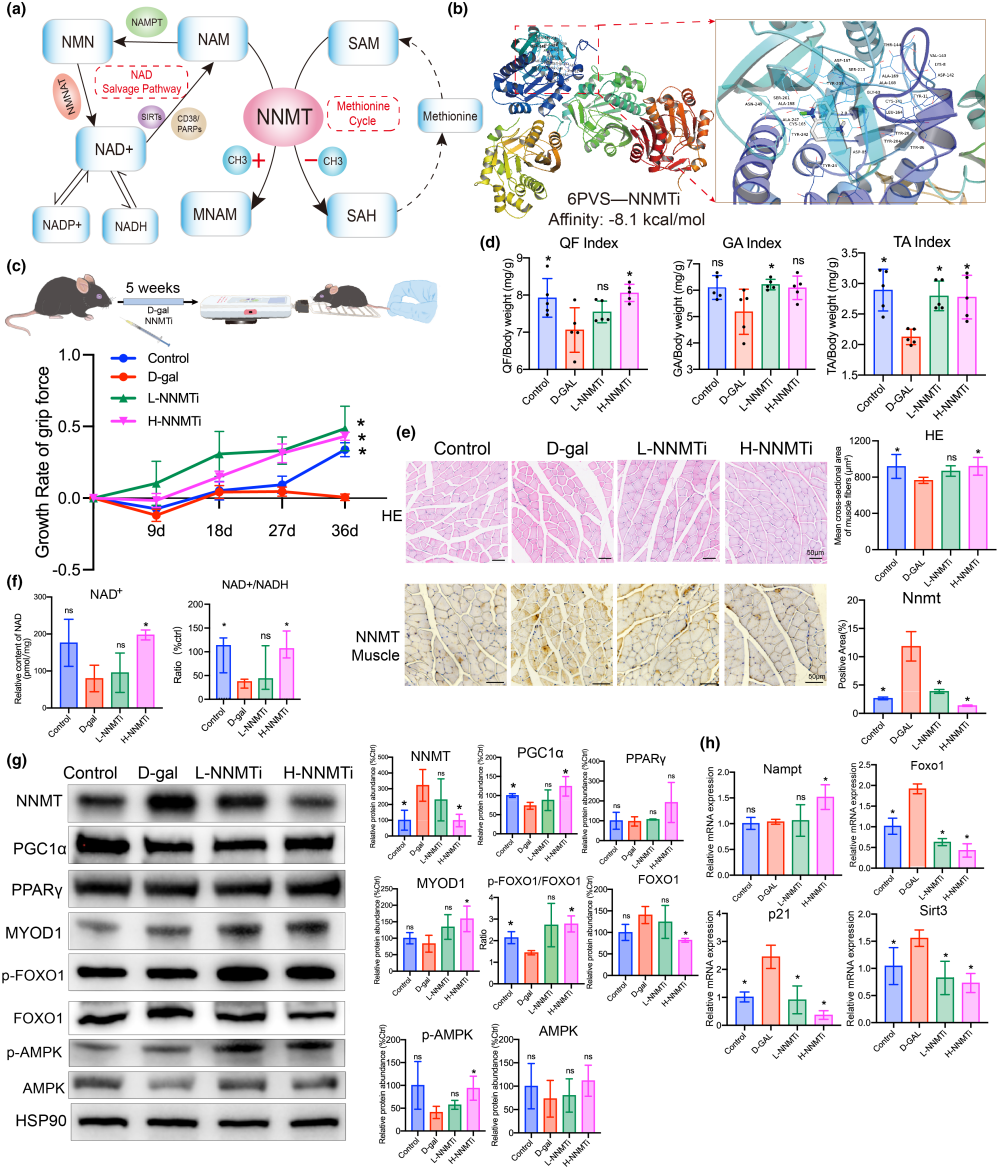
Effects of NNMT inhibition on skeletal muscle structure and function in D‐galactose (D‐gal)‐induced aging mice. (a) Schematic illustration of involvement of NNMT in NAD^+^ salvage pathway and methionine cycle. NAM, nicotinamide; NMN, nicotinamide mononucleotide; MNAM, N1‐methylnicotinamide; SAM, S‐adenosyl methionine; SAH, S‐adenosyl homocysteine. (b) The molecular docking between NNMT protein (PDB ID: 6PVS) and NNMT inhibitor (NNMTi) was conducted using AutoDock vina method, and the docking image was presented using PyMoL (version 1.7.2.1). (c) D‐gal‐induced aging mouse model and the effects of NNMTi treatment on the growth rate of grip force of mice. Low dose of NNMTi treatment, L‐NNMTi; high dose of NNMTi treatment, H‐NNMTi. (d) Skeletal muscle mass index (SMI) of lower limb, including SMI of quadriceps femoris (QF), gastrocnemius (GA), and tibialis anterior (TA) muscles. (e) Representative HE staining images of QA muscle in each group. Representative IHC staining images of NNMT in QA muscle tissues of each group. (f) NAD^+^ amount and NAD+/NADH ratio of mouse muscles from each group. (g) Representative images of western blot presented the protein expression levels of NNMT, PGC1α, PPARγ, MYOD1, p‐FOXO1, FOXO1, p‐AMPK and AMPK in muscles of different groups. (h) Relative mRNA expression levels of *Nampt*, *Foxo1*, *p21* and *Sirt3* in muscles of different groups were determined by qPCR. Student *t*‐test or analysis of variance (ANOVA) was conducted for parametric values. Mann–Whitney test was used to analyze nonparametric values. *p < 0.05 versus D‐gal group. ns, not significant.

NNMT plays critical roles in NAD^+^‐related metabolism and methionine cycle pathways (Figure 5a). To investigate the role of NNMT in sarcopenia, we established a mouse model of D‐galactose‐induced aging and treated it with low or high concentrations of NNMT inhibitor (NNMTi). Molecular docking revealed a tight binding (affinity: −8.1 kcal/mol) of NNMTi to the NNMT protein (Figure 5b). We found that mice in the D‐galactose (D‐gal) group exhibited a lower grip index growth rate compared to the control group. However, when treated with NNMTi, this decrease was attenuated (grip index growth rate measured on the 36 day, D‐gal group vs. H‐NNMTi [high concentration of NNMTi] group, 0.0057 vs. 0.4317, *p* < 0.001) (Figure 5c). Particularly, the H‐NNMTi‐treated group had a much higher QF and TA muscle mass index compared to the D‐gal group, indicating improvement in D‐galactose‐induced muscular damage (Figure 5d). Additionally, HE staining showed that the average area of muscle fibers in the H‐NNMTi group was higher than in the D‐gal group (about 1.2 times, *p* < 0.05), and NNMTi treatment improved the deranged structure of the hepatic cord and hepatocyte edema caused by the D‐gal treatment (Figure 5e; Figure S5c). IHC analysis revealed that QF muscles and liver tissues in the D‐gal group exhibited a higher Nnmt expression level compared to the control or H‐NNMTi groups. Moreover, NNMTi treatment decreased the NNMT expression in the QF muscle and liver tissues compared to D‐gal treatment (Figure 5e; Figure S5c). These findings suggest that NNMT plays a significant role in sarcopenia and that its inhibition using NNMT inhibitor may have a potential therapeutic effect in mitigating age‐related muscle decline and improving overall muscular health.

Furthermore, we measured the amount of NAD^+^ in muscles and calculated the NAD^+^/NADH ratios. The H‐NNMTi group exhibited a significantly higher NAD^+^ content in the muscles compared to the D‐gal group, approximately 2.5 times higher (*p* < 0.05). The NAD^+^/NADH ratio in the D‐gal group was lower than that in both the control and H‐NNMTi groups (Figure 5f). Additionally, Western blotting analysis showed that the D‐gal group had higher NNMT expression (about 3.21 times, *p* < 0.05) and lower PGC1α expression (about 0.73 times, *p* < 0.05) compared to the control group. Treatment with H‐NNMTi downregulated the NNMT expression level (approximately 0.43 times, *p* < 0.05), while upregulating the expression levels of PGC1α (approximately 1.70 times, *p* < 0.05) and MYOD1 (approximately 1.91 times, *p* < 0.05) in muscle tissues compared with D‐gal intervention, but did not affect PPARγ expression (Figure 5g). Moreover, the H‐NNMTi group exhibited elevated levels of p‐AMPK expression and p‐FOXO1/FOXO1 ratio compared to the D‐gal group, while displaying lower FOXO1 expression levels. However, there were no significant differences in protein expression levels of AMPK among the groups (Figure 5g). Quantitative real‐time PCR (qPCR) assays revealed that the mRNA expression level of nicotinamide phosphoribosyltransferase (*Nampt*) rather than nicotinamide nucleotide adenylyltransferase 1 (*Nmnat1*) in the H‐NNMTi group was higher than that of the D‐gal group (0.48‐fold, *p* = 0.03). Notably, D‐gal treatment upregulated the mRNA expression levels of Foxo1 (0.90‐fold, *p* < 0.01), P21 (1.44‐fold, *p* < 0.01), and Sirt3 (0.51‐fold, *p* = 0.03) compared to the control group, whereas treatment with a high NNMTi reversed the expression of these genes. However, no significant differences in Sirt1 and Foxo3 mRNA expression levels were observed between the groups (Figure 5h; Figure S5d).

### 3.5 Targeted inhibition of NNMT in the prevention and treatment of sarcopenia

**FIGURE 6:**
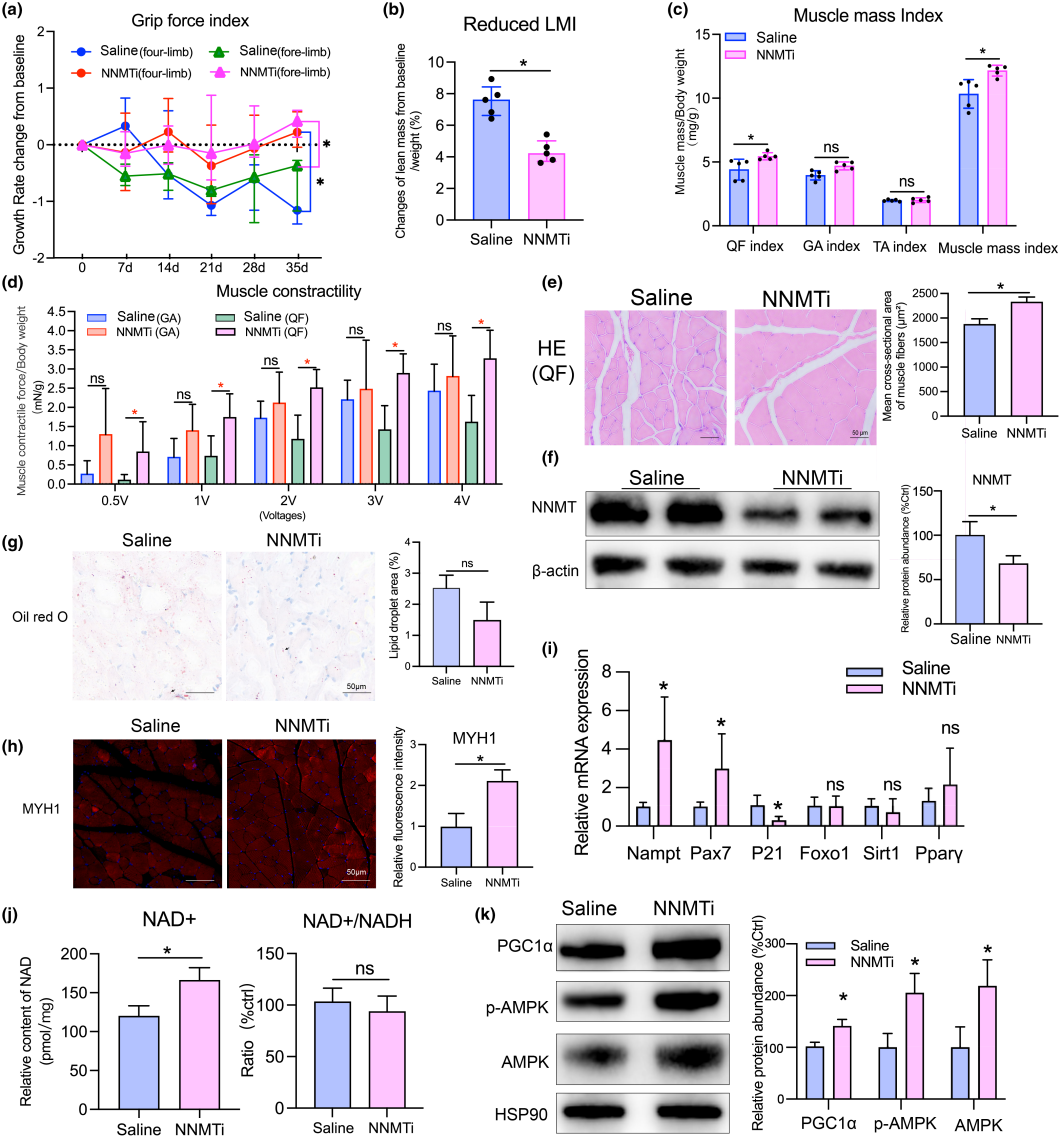
Preventive and therapeutic effect of NNMT inhibition on aging‐related muscle injury. (a) The effects of NNMTi treatment on the growth rate of grip force of old mice. (b) The changes of lean mass index (lean mass/body weight) from baseline to the end of the intervention detected by DXA. (c) Skeletal mass index of QF, GA, and TA of the left lower limbs. (d) Maximum muscle contraction force of QF and GA by electrical stimulation at different voltage. (e) Representative HE staining images of QA muscle tissues. (f) Representative images of western blot presented the protein expression levels of NNMT and β‐Actin in QF muscle tissues. (g–h) Representative images of Oil red O (ORO) staining (g) and immunofluorescence (IF) staining of MYH1. Percentage of ORO‐stained area and IF area and density were analyzed using ImageJ. (i) Relative mRNA expression levels of *Nampt*, *Pax7*, *Foxo1*, *p21*, *Sirt1*, and *Pparγ* in muscles. (j) NAD^+^ amount and NAD^+^/NADH ratio of old mouse muscles. (k) Representative images of western blot presented the protein expression levels of PGC1α, p‐AMPK, and AMPK. Student *t*‐test or analysis of variance (ANOVA) was conducted for parametric values. Mann–Whitney test was used to analyze nonparametric values. *p < 0.05 versus D‐gal group. ns, not significant.

To further elucidate the regulatory function of NNMT during skeletal muscle aging, 19‐month‐old mice were administered NNMTi. The results indicated that inhibition of NNMT slowed down the decline in the forelimb and four‐limb grip strength index in old mice (Figure 6a). Lean body mass and fat mass were assessed using DXA before and after NNMTi interventions, and the results revealed that NNMT inhibition may alleviate the decrease in the lean mass index (LMI) (reduced LMI, Saline group vs. NNMTi group, 7.53% vs. 4.34%, *p* < 0.05). However, there was no notable impact on the alteration of the fat mass index (fat mass/body weight) across the groups (Figure 6b; Figure S6a,b). Additionally, the NNMTi group had a higher QF mass index (Figure 6c). Muscle contractility analysis demonstrated that the contraction force of QF and GA increased with the escalating stimulation voltage. The contraction force of QF in the NNMTi group was significantly greater than that in the saline group (about 2.02 times of the control group when the stimulation voltage was 4V, *p* < 0.05), whereas the contraction force of GA did not exhibit a significant difference between the groups (Figure 6d).

In comparison to the saline group, the NNMTi group exhibited lower levels of NNMT protein (about 0.7 times, *p* < 0.05) in the QF muscles, and a larger average muscle fiber area (about 1.24 times, *p* < 0.01) (Figure 6e,f). Oil red O staining did not reveal any discernible differences in the lipid droplet area between the two groups (Figure 6g). Myosin heavy chain 1 (MYH1), a marker protein indicative of rapid muscle fiber subtype IIX (rapid glycolysis) (Long et al., 2022), was found to have a higher fluorescence density in the QF muscles of the NNMTi group compared to the saline group based on immunofluorescence staining. However, there was no notable disparity in mRNA levels of Myosin heavy chain 2 (*Myh2*) and 7 (*Myh7*) between the two groups (Figure 6h; Figure S6d). In contrast to the saline group, the NNMTi group had higher mRNA levels of *Nampt*, *Pax7*, and lower expression levels of *p21*. Conversely, the mRNA levels of *Foxo1*, *Sirt1*, and *Pparγ* did not display a significant variance (Figure 6i). Analysis of NAD^+^ content revealed that the NNMTi group demonstrated a higher NAD^+^ level than the saline group, with no significant distinction in the NAD^+^/NADH ration between two groups (Figure 6j). Western blotting analysis suggested that the NNMTi group exhibited increased PGC1α, p‐AMPK, and AMPK protein expression levels (Figure 6k). Additionally, we detected the reactive oxygen species (ROS) production of QF muscle tissues. The fluorescence intensity revealed that although the NNMTi group had a lower amount of ROS, no statistical difference was found between the groups. qPCR analysis suggested that the NNMTi group exhibited higher mRNA levels of nuclear respiratory factor (*Nrf2*), whereas the mRNA levels of heme oxygenase 1 (*Ho‐1*) showed no significant variations across the groups (Figure S6c,d).

## 4 DISCUSSION

Sarcopenia is a progressive, generalized skeletal muscle disease closely associated with the aging process. Its pathogenesis involves multifaceted interactions, including genetic inheritance and environmental factors. Researchers have focused on various predisposing factors such as oxidative stress, mitochondrial dysfunction, and altered endocrine hormone levels. These factors often coexist with aging, mutually exacerbating each other and adding to the complexity of sarcopenia's pathogenesis (Sayer & Cruz‐Jentoft, 2022). Skeletal muscle plays a crucial role in hormonal and neural regulation, functioning as an important endocrine organ. Therefore, alterations in the metabolic microenvironment, such as disorders of glucose, lipid, protein, and/or energy metabolism, usually occur during pre‐sarcopenia or throughout the progression of sarcopenia (Daily & Park, 2022). This underscores the potential promise of exploring sarcopenia mechanisms and intervention strategies through the lens of metabolic regulation. This study explored the relationship between dysregulated metabolism and sarcopenia and identified critical metabolic regulators associated with sarcopenia. Subsequently, the effect of inhibiting target genes on skeletal muscle damage was tested in both D‐galactose‐treated mouse model and naturally aging mouse model. The findings suggest that NNMT is involved in metabolic disorders associated with sarcopenia and could serve as a diagnostic marker for the condition. Targeted inhibition of NNMT in a mouse model improved age‐related declines in muscle strength and muscle mass.

This study found that the DEGs of sarcopenia acquired from Dataset_1 were closely related to metabolism‐related diseases like fatty liver, obesity, and type 2 diabetes. These DEGs were found to be enriched in important signaling pathways, including PPAR, AMPK, and TGFβ. A systematic review revealed a significant positive correlation between sarcopenia and metabolic syndrome (MS) in middle‐aged and older non‐obese adults (OR = 2.01, 95% CI, 1.63–2.47) (Zhang et al., 2018). Notably, glucose and lipid metabolism disorders in patients with MS are highly susceptible to the induction of fatty liver disease, type 2 diabetes, and sarcopenia. Hence, dysregulated metabolism is a trigger and vital pathological change in sarcopenia. Fat infiltration in muscle tissues, intramyocellular lipid deposition, and insulin resistance are commonly observed during sarcopenia progression (Al Saedi et al., 2022). To identify metabolic target genes, we integrated the metabolic gene set with LASSO regression, randomForest, SVM‐RFE, and ROC analyses, and validated the results in Dataset_2. Among the candidate genes, NNMT was closely associated with muscle aging and upregulated in the muscles of patients with sarcopenia, suggesting its potential value as a diagnostic marker for sarcopenia.

NNMT is an important metabolic enzyme in both the NAD^+^ salvage and methionine cycle pathways (Figure 5a), that affects cellular metabolism and methylation levels by regulating the NAD^+^/NADH and SAM/S‐adenosyl homocysteine (SAH) ratios in cells (Pissios, 2017). It is upregulated in white adipose tissue of type 2 diabetes patients and negatively correlated with insulin sensitivity in insulin‐resistance individuals (Kannt et al., 2015). Additionally, NNMT overactivation has been linked to the promotion of fatty liver development by impacting the NAD^+^/Sirt3 axis (Komatsu et al., 2018). This study found that the DEGs of sarcopenia were enriched in multiple metabolic processes including fatty acid metabolism, glycerolipid metabolism, cysteine and methionine metabolism, and glycolysis/gluconeogenesis (Dataset_3). These processes are involved in several energy metabolism‐related molecular functions, such as aldehyde dehydrogenase (NAD^+^/NADH) activity, carbohydrate binding, and superoxide‐generating NADPH oxidase activator activity. Given its involvement in the regulation of these pathways, NNMT emerges as a critical regulator that contributes to the impairment of energy and lipid metabolism in sarcopenia.

NAD^+^ is an auxiliary agent for hundreds of enzymatic reactions in the body, but its cellular content tends to decrease with aging. This decline in NAD^+^ levels can promote the occurrence and development of age‐related diseases by inducing metabolic dysfunction, DNA repair disorders, inflammation, and neurodegeneration (Covarrubias et al., 2021). A previous study showed that NAD^+^ biosynthesis was reduced in patients with sarcopenia (Migliavacca et al., 2019). NNMT, a key player in NAD^+^ regulation, is thus implicated in modulating NAD^+^‐related pathways involved in the muscle aging process. The analysis of multiple datasets of skeletal muscle from several previous studies shed light on the role of NNMT. Its expression level was found to be associated with age and metabolic status, being significantly higher in metabolically disordered old skeletal muscle compared to non‐metabolically disordered old or young skeletal muscle (Gueugneau et al., 2021). Moreover, inflammatory stress and lipid metabolism‐related injury could up‐regulate the expression of NNMT in skeletal muscle cells, while the treatment of NAD^+^ intermediate NMN decreased the expression level of NNMT in muscle and improved skeletal muscle energy metabolism (Mills et al., 2016; Williams et al., 2020). This study found that patients with sarcopenia had a lower average area of muscle fibers compared to the control population, and were accompanied by increased apoptosis levels of myocytes, and upregulated indicators of muscular atrophy. Notably, the metabolic regulator NNMT was also upregulated in patients with sarcopenia (Figure 4i).

Various inhibitors have been developed for the research and exploration of metabolic diseases based on the physiological functions and main regulatory pathways of NNMT (Gao et al., 2021). Notably, a study demonstrated that inhibiting NNMT led to the upregulation of Pax7 expression, promoting the repair and regeneration of muscles in old mice with acute muscle injury (Neelakantan et al., 2019). This study selected the inhibitor NNMTi for further validation. When both D‐gal‐induced aging mice and naturally aging mice were administered NNMTi, the results, including grip index tests, lower limb muscle mass index data, and changes in body lean body mass detected by DXA, indicated that the downregulation of NNMT expression played a role in mitigating muscle strength and mass decline. Additionally, NNMTi treatment appeared to alleviate the reduction in the muscle fiber cross‐sectional area and muscle contraction force in the aging process of QF muscles in old mice and also improved d‐gal‐induced injury and edema in liver tissue. Nevertheless, the NNMTi treatment did not yield statistically significant effects on the changes in fat mass and fat infiltration area of skeletal muscle in old mice. Interestingly, treatment with NNMTi in two aging mouse models led to an increase in Nampt expression levels and NAD^+^ content. Previous studies showed that Nampt upregulation improves glycolipid metabolism and NAD^+^‐related mitochondrial function in the treatment of muscle metabolic disorder atrophy (Manickam et al., 2022). Therefore, the metabolic regulation produced by NNMT inhibition is closely related to the upregulation of Nampt and NAD^+^. Moreover, the levels of Pgc1α and p‐AMPK in the H‐NNMTi group or NNMTi group were found to be higher than those in the model group or saline group, while Pparγ did not exhibit significant changes among the groups. This suggests that the mechanism of NNMT regulating muscle energy metabolism was related to the NAD^+^/AMPK axis. The QF muscle exhibits vigorous and swift somatic activity and is classified as a “white glycolytic muscle” owing to its abundance of type IIX muscle fibers (Long et al., 2022). Our investigation revealed a significant increase in MYH1 expression in the QF muscles of the NNMTi group compared to the saline group, suggesting a possible augmentation in glycolytic muscle fiber content in elderly mice after NNMTi treatment. Nevertheless, further studies are necessary to explore the role of the NNMT gene in the conversion between fast and slow muscle fiber types. Additionally, NNMTi can promote the repair of muscle growth, also potentially inhibit muscle cell aging and atrophy. Inhibition of NNMT in older mice led to an upregulation of *Pax7* expression and a downregulation of *p21* expression in QF muscles.

This study encountered several limitations that warrant acknowledgment. First, Dataset_1 included muscle tissues from older adults with or without sarcopenia, while Dataset_2 contained samples from both young and old adults. Despite this discrepancy, NNMT demonstrated good diagnostic efficacy for sarcopenia. However, it is essential to recognize this difference in sample sources as a potential confounding factor in the interpretation of the results. Second, Dataset_3, which had a sample source consistent with Dataset_1, had a relatively small sample size, leading to less robust validation of NNMT's diagnostic efficacy for sarcopenia. To strengthen the reliability of the findings, we plan to increase the sample size in future studies for more robust validation. Third, the regulatory effects of NNMT on muscle metabolic disorders were validated in a D‐galactose‐induced aging mouse model and a naturally aging mouse model. D‐galactose is a crucial compound in age‐related diseases and has been utilized in constructing models for various aging‐related conditions, including sarcopenia (Azman & Zakaria, 2019). Research has confirmed that D‐galactose intervention induces metabolic dysregulation in mice, resulting in skeletal muscle atrophy and decreased muscle strength (Wang et al., 2023; Wu et al., 2022). Moreover, the role of NNMT inhibition in preventing age‐related muscle damage was explored in a natural aging mouse model. However, the mouse models of sarcopenia established based on data of lean mass index, grip force index, and physical function, can provide a more effective means of investigating the mechanism of NNMT metabolic pathway intervention in aging‐related skeletal muscle damage. For enhanced translational potential in research, additional studies using diverse animal models and potential human clinical trials would be more beneficial. Last, in‐depth exploration of molecular mechanisms focused on AMPK and PPAR pathways based on omics analysis. Although these pathways are relevant and interesting, it is crucial to recognize that NNMT may have other molecular mechanisms influencing sarcopenia that remain undiscovered. Future research should explore and elucidate these other potential mechanisms to gain a comprehensive understanding of NNMT's action in the context of sarcopenia.

In conclusion, this study highlights the significance of dysregulated metabolism as a critical factor in the pathogenesis of sarcopenia. Notably, NNMT has been identified as a potential diagnostic marker and a potential therapeutic target for sarcopenia. Targeted inhibition of NNMT could improve energy metabolism in skeletal muscle via regulating the NAD^+^/AMPK axis, thereby alleviating muscle atrophy and promoting muscle damage repair. The findings from this research provide a novel direction for exploring clinical prevention and treatment strategies for sarcopenia.

## AUTHOR CONTRIBUTIONS

JRY and RL designed this study and RL drafted the manuscript. JRY, YL, and DMX revised the manuscript. RL, QX, and MD performed the experiments, collected the data, and contributed equally to this article. QHS and TPL conducted the statistical analysis. All authors read and approved the final manuscript.

## CONFLICT OF INTEREST STATEMENT

All authors declare no conflict of interest.

## Supporting information


Data S1.

Figures S1–S6.

## Data Availability

All data needed to evaluate the conclusions in this study are presented in this article and its additional files. All data analyzed or generated during the study are available upon reasonable request.
