# Supplementary material for: Identification of nicotinamide N‐methyltransferase as a promising therapeutic target for sarcopenia
Source: Aging Cell. 2024 Jun 5;23(9):e14236. doi: 10.1111/acel.14236 (PMC11488295; doi:10.1111/acel.14236)
Supplement: Supplementary file 1 — Data S1. [file ACEL-23-e14236-s002.docx]

Supporting information 1

1. Detailed information of datasets analyzed in the study

Table S1. Information of dataset

|  | Data information | Database | Data type | Sample information/Gene number | References  (PMID number) |
| --- | --- | --- | --- | --- | --- |
| Dataset_1 | GSE111006  (Skeletal muscles) | GEO database | Expression profiling by high throughput sequencing | Normal samples：28  Sarcopenia samples：4  (Sarcopenia was diagnosed based on EWGSOP algorithm). Total lean mass was measured by DXA (Hologic Discovery, software version 12.5). | 31862890 |
|  | GSE111010  (Skeletal muscles) | GEO database |  | Normal samples：14  Sarcopenia samples：9  (Sarcopenia was diagnosed based on EWGSOP algorithm). Total lean mass was measured by DXA (GE Lunar Prodigy) Advance, Software: Encore 2011, Version 13.60.033). | 31862890 |
|  | GSE111016  (Skeletal muscles) | GEO database |  | Normal samples：20  Sarcopenia samples：20  (Sarcopenia was diagnosed based on AWGSOP definition). Total lean mass was measured through DXA scanning (APEX Software version 4.0.1, Discovery Wi DXA system). | 31862890 |
| Dataset_2 | GSE38718  (Skeletal muscles) | GEO database  (Platforms: GPL570) | Expression profiling by array | 19~28 years：14  65~76 years：8 | 23418191 |
|  | GSE8479  (Skeletal muscles) | GEO database  (Platforms: GPL2700) |  | Young muscle samples：26  Aged muscle samples：25 | 17520024 |
| Dataset_3 | RNA-Sequencing  (Skeletal muscles) | / | High throughput sequencing | Normal samples：5  Sarcopenia samples：5  Total lean mass was measured by bioelectrical impedance analyzer, (InBody S10, Korea). | / |
|  | GSE164471  (Skeletal muscles) | GEO database | High throughput sequencing | 20-29 years: 8  30-39 years: 9  40-49 years: 6  50-59 years: 8  60-69 years: 8  70-79 years: 9  Over 80 years: 5 | 33795677 |
|  | GSE175495  (Skeletal muscles) | GEO database | High throughput sequencing | 20-35 years: 12  60-85 years: 12 | 33895996 |
|  | PXD011967  (Skeletal muscles) | ProteomeXchange (PX) Consortium  (https://www.proteomexchange.org/) | Nano LC-MS/MS Analyses | 20-40 years: 9  60-86 years: 14 | 30971946 |
|  | GSE136344  (Skeletal muscles) | GEO database | Expression profiling by array | Young samples: 11  Old samples: 12  Metabolic syndrome samples: 7 | 33921590 |
|  | GSE126101  (human primary muscle cells) | GEO database | High throughput sequencing | Treated with palmitate or TNFa | 32483258 |
|  | GSE85718  (Skeletal muscles, Liver, White adipose tissue) | GEO database | Expression profiling by array | Control :4  NMN treatment (6 months): 4  NMN treatment (12 months): 4 | 28068222 |
| Metabolism related gene set | https://pathcards.genecards.org/ | PathCards (PATHWAY UNIFICATION DATABASE) | Gene set | Metabolism SuperPath: 2121 genes (Search date: July 20, 2023) | / |
| The Genotype-Tissue Expression (GTEx) Portal | <https://www.gtexportal.org/home/>  (Skeletal muscles) | The Genotype Tissue Expression (GTEx) Project | RNA sequencing (bulk RNA-seq) | Healthy skeletal muscle samples with different ages: 803 | 25954001,  26484571 |

LC/MS: liquid chromatography/ mass spectrometry; NMN: nicotinamide mononucleotide

1. Additional materials and methods

2.1 Muscle tissue collection

Muscle tissue samples in the present study were collected at West China Hospital of Sichuan University. The participants included in our study were patients undergoing general anesthesia for the excision of benign tumors, such as lateral femoral hemangioma, lateral femoral lipoma, femoral neck osteochondroma, and lateral femoral neurofibroma. During the surgical procedure, we collected normal muscle samples from areas adjacent to the diseased tissue, more than 2 cm away from the tumor site. Specifically, we dissected and exposed the vastus lateralis muscle, isolating approximately 2cm × 1cm × 1cm sections of normal muscle tissue. Following excision, the muscle samples were promptly rinsed with saline solution to remove any surface blood. Using sterile scissors and forceps, we carefully removed fascia and adipose tissues to isolate pure muscle tissue. The samples were then dried on sterile gauze to remove excess moisture, and subsequently divided into 2-3 smaller pieces. These pieces were aliquoted into 2.0 ml cryovials and rapidly freezing in liquid nitrogen and then stored at -80 °C to ensure the integrity of RNA and protein. The procedure of muscle collection was approved by the Ethic Committee on Biomedical Research of West China Hospital of Sichuan University (No: 2020-539). All patients and their families were informed and signed an informed consent form. Information of sample collection is presented as follows:

Inclusion Criteria: (1) Age 60 years or older. (2) Absence of severe cognitive impairment, which was determined by standardized cognitive assessment tools, such as Mini-Mental State Examination (MMSE). (3) Ambulatory and relatively independent in activities of daily living. (4) Willingness and ability to adhere to study protocols and attend scheduled assessments. (5) Provision of informed consent to participate in the study.

Exclusion Criteria: (1) Diseases that would cause changes in body composition in a short period of time, such as fever, diarrhea and edema; patients with neuromuscular diseases, including myathenia gravis, polymyositis, periodic paralysis, peripheral neuropathy, Duchenne and Becker muscular dystrophy; other conditions that affect the measurement of body composition, such as severe spinal deformity and having a cardiac pacemaker or other metallic medical devices installed. (2) Recent history of acute illness or hospitalization within the past three months. (3) End-stage renal disease or severe hepatic dysfunction. (4) History of malignancy within the past five years. (5) Any contraindications to physical activity or resistance training. (6) Use of medications known to significantly affect muscle mass or function, such as systemic corticosteroids. (7) Uncontrolled psychiatric disorders or substance abuse.

Diagnostic criteria for sarcopenia: Sarcopenia was diagnosed in accordance with the Asian Working Group for Sarcopenia criteria in 2019. (a) Maximum handgrip strength (both hands) ＜28 kg in male and ＜18 kg in female. (b) Skeletal muscle mass index (SMI) measured by a bioelectrical impedance analyzer is less than 7.0 kg/m^2^ in male and 5.7 kg/m^2^ in female. Sarcopenia must meet both criteria (a) and (b). Non-sarcopenia meets neither (a) nor (b).

3) The basic clinical data are presented in Table S2.

Table S2. Clinical information

| Term | Non-sarcopenia  (n = 5) | Sarcopenia  (n = 5) | P value |
| --- | --- | --- | --- |
| Age (years), Mean (SD) | 70.40 (5.03) | 66.80 (5.22) | 0.299 |
| Sex, Female (%) | 3 (60) | 4 (80) | / |
| SMI (Kg/m^2^) | 6.74 (0.88) | 5.00 (0.58) | 0.006* |
| Grip (Kg) | 22.68 (4.95) | 13.98 (2.92) | 0.010 |
| Height (cm) | 158.00 (11.40) | 148.80 (4.56) | 0.132 |
| Weight (kg) | 60.60 (12.22) | 44.80 (5.26) | 0.029 |
| BMI (kg/m^2^) | 24.42 (5.08) | 20.32 (3.02) | 0.10 |
| Total protein (g/L) | 73.72 (5.40) | 65.30 (9.45) | 0.122 |
| Albumin (g/L) | 45.62 (4.56) | 38.04 (5.54) | 0.046 |
| Blood glucose (mmol/L) | 5.75 (1.32) | 6.09 (1.54) | 0.716 |
| Cholesterol (mmol/L) | 5.26 (0.86) | 4.22 (0.43) | 0.042 |
| Triglyceride (mmol/L) | 1.66 (0.96) | 1.24 (0.30) | 0.377 |
| LDL (mmol/L) | 3.16 (0.63) | 2.46 (0.37) | 0.064 |
| HDL (mmol/L) | 1.51 (0.40) | 1.24 (0.16) | 0.188 |

SMI: skeletal muscle mass index; BMI: body mass index; LDL: low-density lipoprotein; HDL: high-density lipoprotein.

2.2 Hematoxylin and eosin (HE) and immunohistochemistry (IHC) staining

Tissues were embedded in paraffin and used for HE and IHC staining according to routine protocols. For HE staining, the sections were immersed in Environmental Friendly Dewaxing (Servicebio, China), anhydrous ethanol and 75% Ethyl alcohol in sequence for dewaxing and hydration. Subsequently, hematoxylin staining and esodin staining were performed as the procedures, followed by dehydration and sealing. For IHC, the sections were immersed in 3% hydrogen peroxide solution to block endogenous peroxidase, uniformly covered using 3% BSA, and incubated with the primary antibodies (1:100 or 1:200) over night at 4℃, including anti-MURF1 from HuaAn Biotechnology (Hangzhou HuaAn Biotechnology Co., Ltd., China) and anti-NNMT from Abcam (Abcam, USA). After incubating with the secondary antibody of the corresponding species of the primary antibody, the slides were washed, dried and colored using DAB chromogenic reagent for histochemical kit (Servicebio, China). Finally, the nucleis of the tissues were restained. All sections were observed using a Nikon microscope (Nikon, Japan), and the images were analyzed using Image J software (Image J, USA).

2.3 Quantitative real-time PCR (qPCR)

Total RNA was extracted using Eastep^®^ Super Total RNA Extraction Kit (Promega, Shanghai, China) following the manufacturer’s instructions. cDNA synthesis was performed using HiScript^®^ III RT SuperMix for qPCR (Vazyme Biotech Co. Ltd., China). The primer sequences were listed in Table S3. The qPCR assay was conducted on QuantStudio3 (ThermoFisher, USA) using ChamQ Universal SYBR qPCR Master Mix (Vazyme). The mRNA expression levels of genes were assessed using a 2^-△△Ct^ method and normalized to GAPDH or Beta-actin.

Table S3. The list of primers of the genes for qRT-PCR

| Gene name | Forward Primer | Reverse Primer |
| --- | --- | --- |

| Nampt | CTGTGTCTGTGGTCAGCGATAGC | GGTCTGATGATTAGTGGTGCCTCTG |
| --- | --- | --- |
| Nmnat1 | CCCAAGAGCCCAAACCAACAGG | CCGAGTGATACAGATGAGCCCAAAG |
| Foxo1 | AGTGGATGGTGAAGAGCGTG | GAAGGGACAGATTGTGGCGA |
| Foxo3 | CGTTCCTGAAGGGAAGGAGC | CGACTCTGTGGCTCGAACTC |
| P21 | GGTGGTGGAGACCTGATGATAC | TATTCTGCTGGCAAAGTGGGA |
| Sirt1 | CCAGACCTCCCAGACCCTCAAG | GTGACACAGAGACGGCTGGAAC |
| Sirt3 | GCTGCTTCTGCGGCTCTATACAC | CAAAGGTCCCGTGGGCTTCAAC |
| GAPDH | GGTTGTCTCCTGCGACTTCA | TGGTCCAGGGTTTCTTACTCC |
| β-actin | GTGACGTTGACATCCGTAAAGA | GTAACAGTCCGCCTAGAAGCAC |
| Pax7 | AGTATGGCCAAACTGCTGTTGAT | GTAGGCTTGTCCCGTTTCCA |
| Myh2 | TCAGGCTTCAGGATTTGGTGG | CTTGCGGAACTTGGATAGATTTG |
| Myh7 | CCCAGAAACAAGTGAAGAGCCT | GTTCCACGATGGCGATGTTC |
| Nrf2 | TGTCTTAATACCGAAAACAAGCAGC | GACCACAGTTGCCCACTTCTTTT |
| Ho-1 | GCTAAGACCGCCTTCCTGCT | ACGAAGTGACGCCATCTGTGA |
| Pparγ | GACCACTCGCATTCCTTTGACA | ATCGCACTTTGGTATTCTTGGA |

2.4 Western blot

Muscle tissues were washed by cold PBS twice, homogenized in RIPA buffer containing proteinase and phosphatase inhibitors (Beyotime, Shanghai,China), and lysed on ice for 25 minutes. After centrifugation, the supernatant was used for concentration detection by a BCA kit (Beyotime). The western blot assay was conducted as usual. Briefly, equal amounts of tissue proteins were separated by 10% SDS-PAGE gels (EpiZyme, Shanghai, China) and transferred onto PVDF membranes (Millipore, USA). After being blocked in 5% milk-TBST, the membranes were incubated the primary antibodies (1:500 or 1:1000), including anti-PGC1α, anti-HSP90, anti-PPARγ, anti-MYOD1, anti-AMPK, anti-FOXO1, anti-GAPDH and anti-β-actin from HuaAn Biotechnology Co., Ltd. (Shanghai, China), anti-phospho-AMPK (T183/172) and anti-phospho-FOXO1 (S256) from ImmunoWay (Plano, USA), and anti-Nnmt (Abcam, USA). Subsequently, the membranes were incubated with species-specific secondary antibodies (1:3000-1:5000, HuaAn, China). The protein bands were exposed using a Chemiluminescent Assay Kit (EpiZyme) and the images were analyzed by Image-Pro Plus software (version 6.0).

2.5 Oil Red O staining

Fresh frozen sections of 10 µm thickness were prepared from tissues by routine protocols. Sections were placed in absolute propylene glycol for 2-5 minutes before staining in pre-warmed Oil Red O solution for 8-10 minutes at 60 ºC. Sections were then placed in 85% propylene glycol for 2-5 minutes, rinsed in two changes of distilled water, and mounted using an aqueous mounting medium. Lipid distribution within the tissue was observed with a Nikon microscope (Nikon, Japan) and analyzed with Image J software (Image J, USA).

2.6 Immunofluorescence staining

Tissues were embedded in paraffin according to routine protocols. Paraffin-embedded tissue sections were handled by sequential immersion in Environmentally Friendly Dewaxing (Servicebio, China), anhydrous ethanol, and 75% ethyl alcohol for dewaxing and hydration. Antigen retrieval was achieved by microwaving sections in citrate buffer (10 mM, pH 6) for 8 minutes, followed by cooling to room temperature. The sections were permeabilized using 0.5% Triton X-100 for 20 minutes and treated with 3% hydrogen peroxide to block endogenous peroxidase activity. Blocking was conducted with 5% BSA for one hour. Sections were incubated overnight at 4°C with primary antibodies anti-Myh1 (1:500; Proteintech, USA). Sections were then washed and incubated with species-specific fluorophore-conjugated secondary antibodies (1:500) for one hour in the dark at room temperature. From this step onward, all procedures were performed under light-protected conditions. After washes, sections were stained with DAPI for 5 minutes to visualize nuclei, and mounted with antifade mounting medium. Imaging was performed using a Nikon fluorescence microscope (Nikon, Japan) with appropriate filters, and the images were analyzed using Image J software (Image J, USA).

2.7 Reactive oxygen species (ROS) assay

The ROS level in the QF muscle was quantified using a DCFH-DA fluorescent probe-based ROS Assay Kit (Beyotime Biotechnology, China), following the manufacturer's instructions. Approximately 15 mg of muscle tissue was weighed and washed with PBS buffer. The tissue was then homogenized in 200 µL of PBS buffer using ultrasonication on ice. After centrifugation at 12,000 rpm for 5 minutes at 4°C, 50 µL of the supernatant was transferred to each well of a black 96-well plate, to which 50 µL of 100 µmol/L DCFH-DA fluorescent probe was subsequently added. Duplicate wells were prepared for each sample, along with the control well containing only the probe. The plate was incubated in the dark at room temperature for 30 minutes. Fluorescence intensity was measured using a microplate reader at an excitation wave length of 485 nm and an emission wave length of 530 nm to assess the ROS level, which was then normalized to tissue weight.

1. Figure legends

**Figure S1.** Principal component analysis presented before and after batch effect removal for GSE111010, GSE111016 and GSE111006 datasets. The results showed that there was no batch effect among three datasets after correction.

**Figure S2.** (a) The Nomogram of 10 featured genes in the sarcopenia diagnosis model based on results of the LASSO regression using Dataset_1. (b) ROC analysis of 10 featured genes for sarcopenia diagnosis.

**Figure S3.** (a) The Nomogram of 13 featured genes in the sarcopenia diagnosis model based on the results of SVM-RFE algorithm using Dataset_1. (b) ROC analysis of 13 featured genes for sarcopenia diagnosis.

**Figure S4.** (a) The Nomogram of 11 featured genes in the sarcopenia diagnosis model based on the results of Random Forest algorithm using Dataset_1. (b) ROC analysis of 11 featured genes for sarcopenia diagnosis.

**Figure S5.** (a) Heatmap showed the expression level of the target genes, including CRES3, NNMT, ADH1B and CYP26B1, according to Dataset_2. Of them, NNMT was significantly upregulated (**P* < 0.05, tested by a linear-based model incorporated in “limma” R package) in sarcopenia. (b) NNMT expression levels across different human tissues (data from GTEx database). (c) Representative HE staining and NNMT IHC staining of the liver from control, D-gal, L-NNMTi and H-NNMTi mice. The NNMT-positive area was calculated using Image J. The significance was analyzed by Mann-Whitney test. **P* < 0.05. (d) qPCR showed the relative mRNA expression level of *Nmnat1, Sirt1* and *Foxo3* among groups. The statistical significance was analyzed by Student t-test. **P* < 0.05, ns, not significant.

**Figure S6.** (a) Representative images of DXA analysis of saline and NNMTi treatment mice. (b) The change of fat percentage and lean mass index in saline and NNMTi treatment mice. The statistical significance was measured by Mann-Whitney test. (c) ROS production was determined by a ROS assay kit and fluorescence microplate reader. The result showed that there was no significant difference of ROS production between saline and NNMTi mice muscle. The statistical significance was measured by Student t-test. (d) qPCR showed the relative mRNA expression level of *Myh2*, *Myh7*, *Nrf2*, and *Ho-1* between groups. Student t-test was conducted for statistical significance analysis. **P* < 0.05, ns, not significant.
