# Supplementary material for: Identification of nicotinamide N‐methyltransferase as a promising therapeutic target for sarcopenia
Source: Aging Cell. 2024 Jun 5;23(9):e14236. doi: 10.1111/acel.14236 (PMC11488295; doi:10.1111/acel.14236)
Supplement: Supplementary file 2 — Figures S1–S6. [file ACEL-23-e14236-s001.docx]

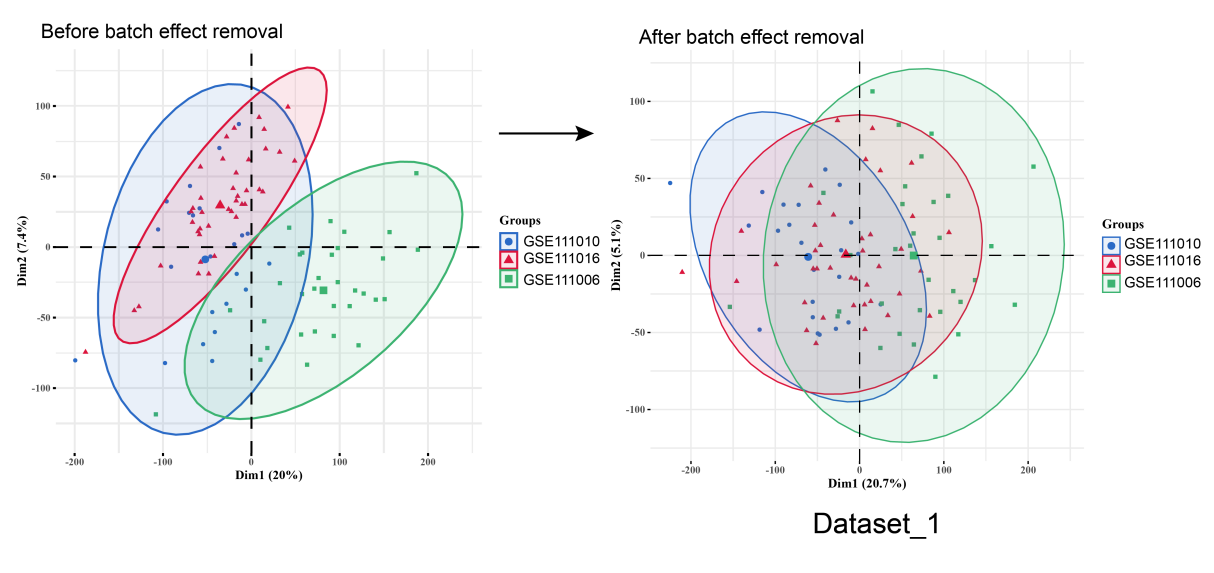


**Figure S1.** Principal component analysis presented before and after batch effect removal for GSE111010, GSE111016 and GSE111006 datasets. The results showed that there was no batch effect among three datasets after correction.


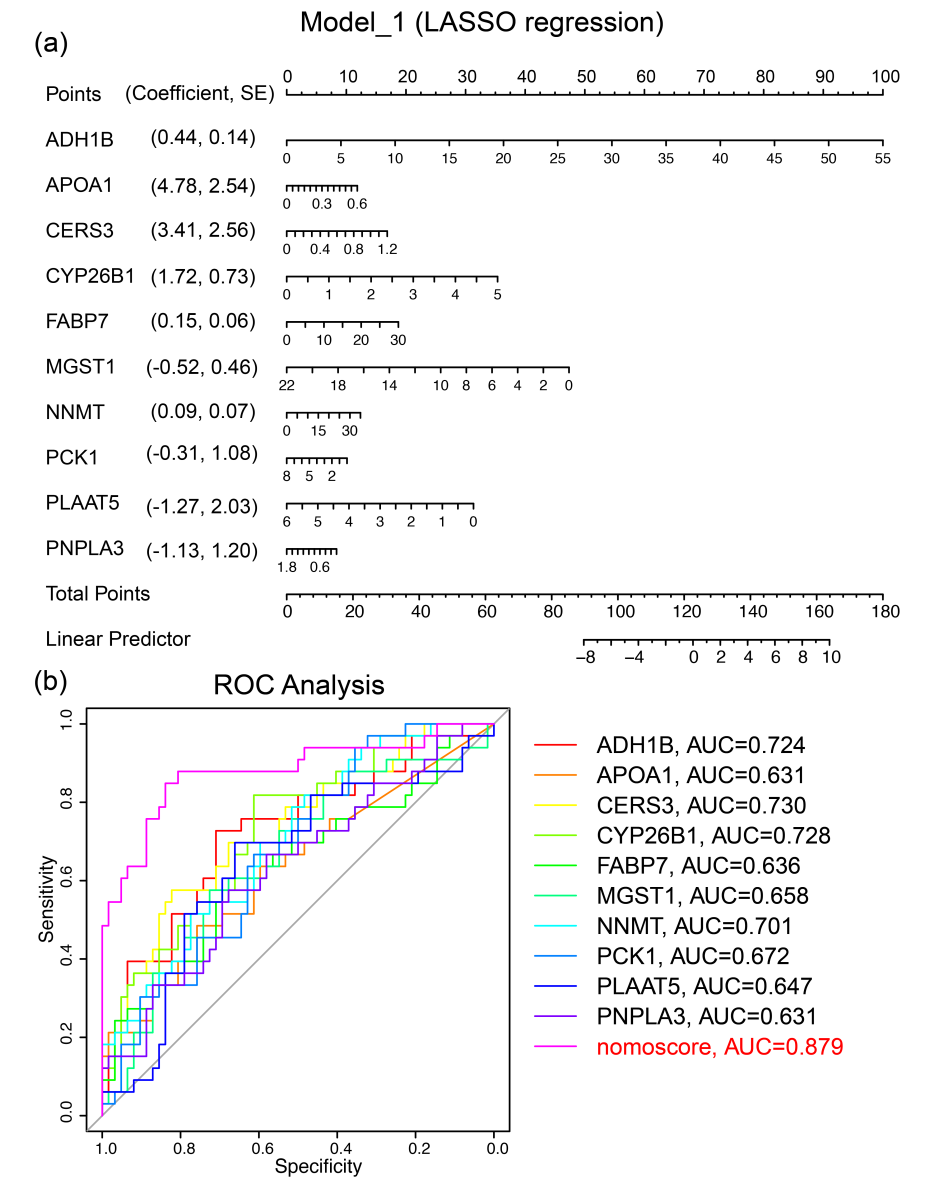


**Figure S2.** (a) The Nomogram of 10 featured genes in the sarcopenia diagnosis model based on results of the LASSO regression using Dataset_1. (b) ROC analysis of 10 featured genes for sarcopenia diagnosis.


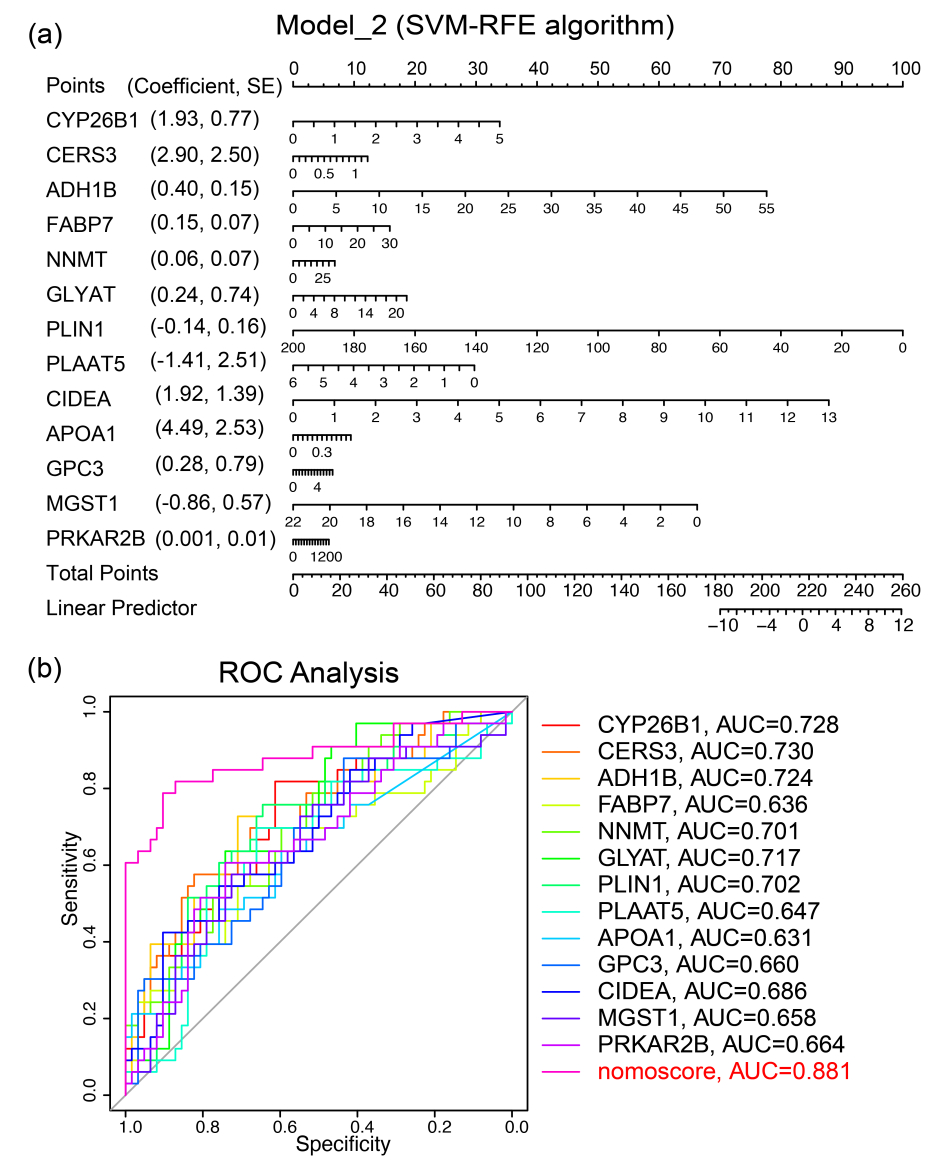


**Figure S3.** (a) The Nomogram of 13 featured genes in the sarcopenia diagnosis model based on the results of SVM-RFE algorithm using Dataset_1. (b) ROC analysis of 13 featured genes for sarcopenia diagnosis.


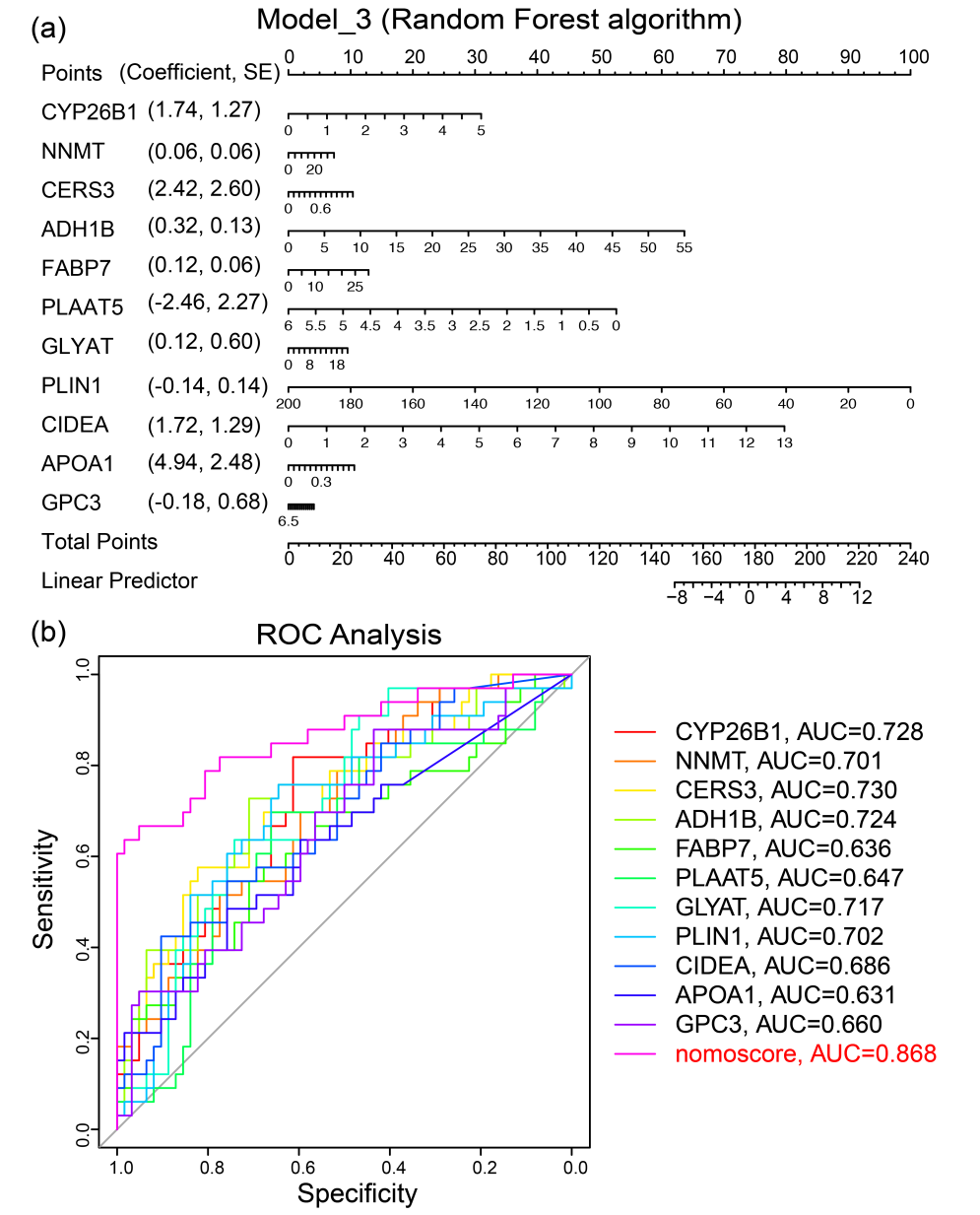


**Figure S4.** (a) The Nomogram of 11 featured genes in the sarcopenia diagnosis model based on the results of Random Forest algorithm using Dataset_1. (b) ROC analysis of 11 featured genes for sarcopenia diagnosis.


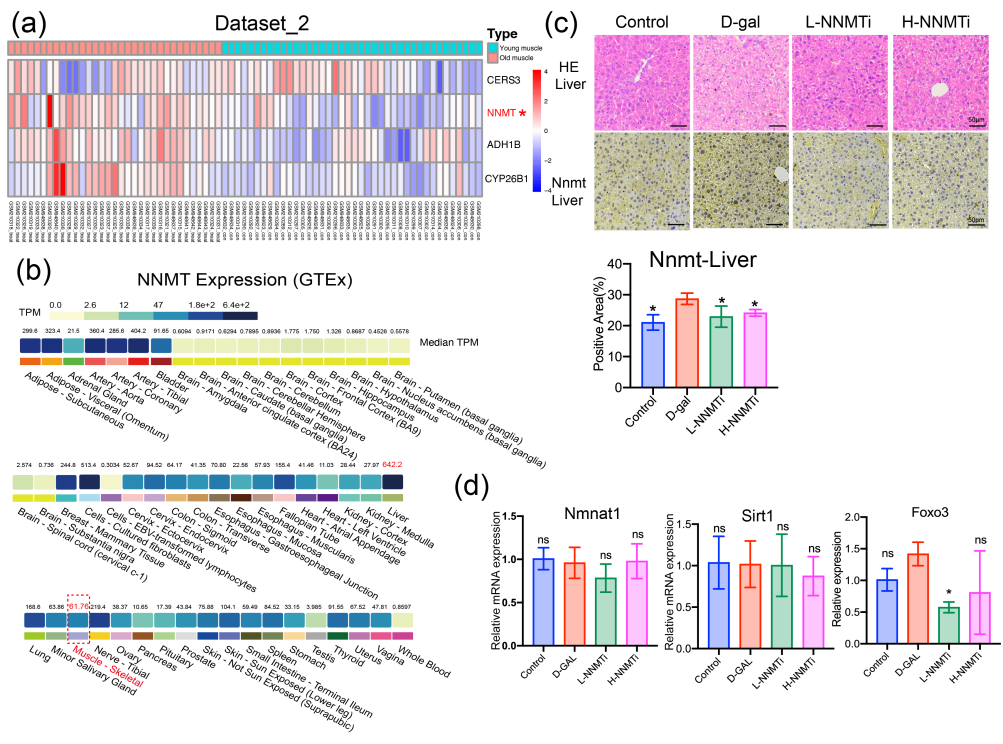


**Figure S5.** (a) Heatmap showed the expression level of the target genes, including CRES3, NNMT, ADH1B and CYP26B1, according to Dataset_2. Of them, NNMT was significantly upregulated (**P* < 0.05, tested by a linear-based model incorporated in “limma” R package) in sarcopenia. (b) NNMT expression levels across different human tissues (data from GTEx database). (c) Representative HE staining and NNMT IHC staining of the liver from control, D-gal, L-NNMTi and H-NNMTi mice. The NNMT-positive area was calculated using Image J. The significance was analyzed by Mann-Whitney test. **P* < 0.05. (d) qPCR showed the relative mRNA expression level of *Nmnat1, Sirt1* and *Foxo3* among groups. The statistical significance was analyzed by Student t-test. **P* < 0.05, ns, not significant.


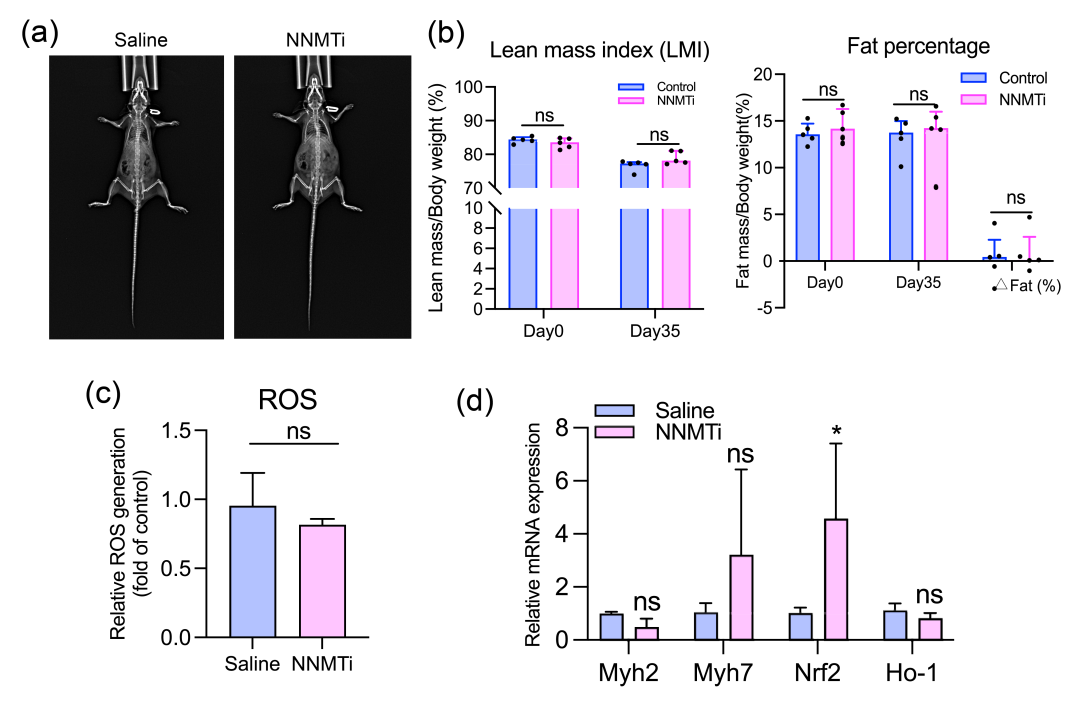


**Figure S6.** (a) Representative images of DXA analysis of saline and NNMTi treatment mice. (b) The change of fat percentage and lean mass index in saline and NNMTi treatment mice. The statistical significance was measured by Mann-Whitney test. (c) ROS production was determined by a ROS assay kit and fluorescence microplate reader. The result showed that there was no significant difference of ROS production between saline and NNMTi mice muscle. The statistical significance was measured by Student t-test. (d) qPCR showed the relative mRNA expression level of *Myh2*, *Myh7*, *Nrf2*, and *Ho-1* between groups. Student t-test was conducted for statistical significance analysis. **P* < 0.05, ns, not significant.
